# Functional brain region-specific neural spheroids for modeling neurological diseases and therapeutics screening

**DOI:** 10.1038/s42003-023-05582-8

**Published:** 2023-11-28

**Authors:** Caroline E. Strong, Jiajing Zhang, Martin Carrasco, Srikanya Kundu, Molly Boutin, Harshad D. Vishwasrao, Jiamin Liu, Angelica Medina, Yu-Chi Chen, Kelli Wilson, Emily M. Lee, Marc Ferrer

**Affiliations:** 1 Division of Preclinical Innovation, National Center for Advancing Translational Sciences (NCATS), National Institutes of Health, 9800 Medical Center Drive, Rockville, MD 20850 USA; 2 Advanced Imaging and Microscopy Resource, National Institutes of Health, Bethesda, MD 20892 USA

**Keywords:** Tissue engineering, High-throughput screening, Neuroscience

## Abstract

3D spheroids have emerged as powerful drug discovery tools given their high-throughput screening (HTS) compatibility. Here, we describe a method for generating functional neural spheroids by cell-aggregation of differentiated human induced pluripotent stem cell (hiPSC)-derived neurons and astrocytes at cell type compositions mimicking specific regions of the human brain. Recordings of intracellular calcium oscillations were used as functional assays, and the utility of this spheroids system was shown through disease modeling, drug testing, and formation of assembloids to model neurocircuitry. As a proof of concept, we generated spheroids incorporating neurons with Alzheimer’s disease-associated alleles, as well as opioid use disorder modeling spheroids induced by chronic treatment of a mu-opioid receptor agonist. We reversed baseline functional deficits in each pilot disease model with clinically approved treatments and showed that assembloid activity can be chemogenetically manipulated. Here, we lay the groundwork for brain region-specific neural spheroids as a robust functional assay platform for HTS studies.

## Abstract (web summary)

The authors describe a method for generating functional neural spheroids and assembloids for disease modeling and drug discovery by cell aggregation of differentiated human iPSC derived neurons and astrocytes at cell type compositions mimicking specific regions of the human brain.

## Introduction

Therapeutic development for neurological diseases has been hindered by a lack of predictable in vitro cellular assays, limited in vivo animal models, and high cost and complexity of clinical trials^1,2^. As a result, less than 10% of clinical candidates to treat neurological diseases are approved by regulatory agencies each year^1–3^. The increase in neurodegenerative disease occurrence and substance use disorders over the last few decades makes it critical to develop technologies to improve the success rate of drug discovery for neurological diseases^4–6^. One way to improve the predictability of assay platforms for drug discovery is to develop functional neural cellular models with high physiological and pathological relevance.

A range of neural models have been developed using induced pluripotent stem cell (iPSC) derived cells, including 2D neural models and 3D neural organoids. While 2D neural models are compatible with high-throughput screening (HTS) assays, 3D neural organoids are generated by an iPSC-differentiation process and include different neuronal and glial cell types and some structural organization^7,8^ and are better at recapitulating in vivo morphology^8^. While organoids acquire greater cellular diversity and some brain-like organization, their complexity, batch-to-batch variation, limited co-culture differentiation, and lengthy maturation times can limit their use for HTS^9^. Cortical neural spheroids, which have been produced from neural stem cells (NSCs) that mature into differentiated excitatory and inhibitory neurons in the spheres^10,11^, maybe a more readily adaptable model for HTS than organoids, but are currently mostly limited to cortical neurons, thus limiting the cell-type composition. The lack of more diverse neuronal subtype populations such as dopaminergic neurons restricts the applications of these spheroids for disease modeling^11–15^.

Here, we describe the development of an HTS-compatible, functional neural spheroid system, referred to here as brain region-specific spheroids, which are assembled by cell aggregation of differentiated human-induced pluripotent stem cell (hiPSC)-derived neurons and astrocytes in a scaffold-free environment. These brain region-specific spheroids can mimic the physiology of distinct brain regions, including the prefrontal cortex (PFC) and ventral tegmental area (VTA) by mixing different neuronal types to mimic the neuronal composition of the different brain regions in vivo. In addition to healthy neural spheroid models, we also describe further application of two diseases including Alzheimer’s disease (AD) using iPSC-derived neurons with genetically engineered, disease-relevant alleles, as well as an opioid use disorder (OUD) neural model induced by chronic treatment with a mu(µ)-opioid receptor (MOR) agonist. We measured intracellular calcium oscillations as an HTS-compatible, functional readout of neural activity, as calcium oscillations have been previously shown to be highly correlated with the electrophysiological properties of neurons^16^, and observed phenotypic differences when comparing “wild-type (Wt)”, or healthy, controls compared to the genetically modified, diseased spheroids. A machine learning classifier model was used to quantify phenotype labeling predictability and showed high accuracy for the AD model (>94%). Furthermore, clinically approved treatments for each disease were used to treat spheroids, and a reversal of deficits was observed for both disease phenotypes. Finally, to address whether we could also extend this technology to neural circuit-specific modeling, we created functional assembloids that are responsive to region-specific chemogenetic manipulations by fusing VTA- and PFC-like spheroids. Together, this study establishes the utility of these brain region-specific neural spheroids through disease and neurocircuitry modeling, and for use as HTS-compatible drug screening platforms.

## Results

### Designer neural spheroids exhibit differential calcium activity profiles depending on neuronal subtype composition

**Fig. 1:**
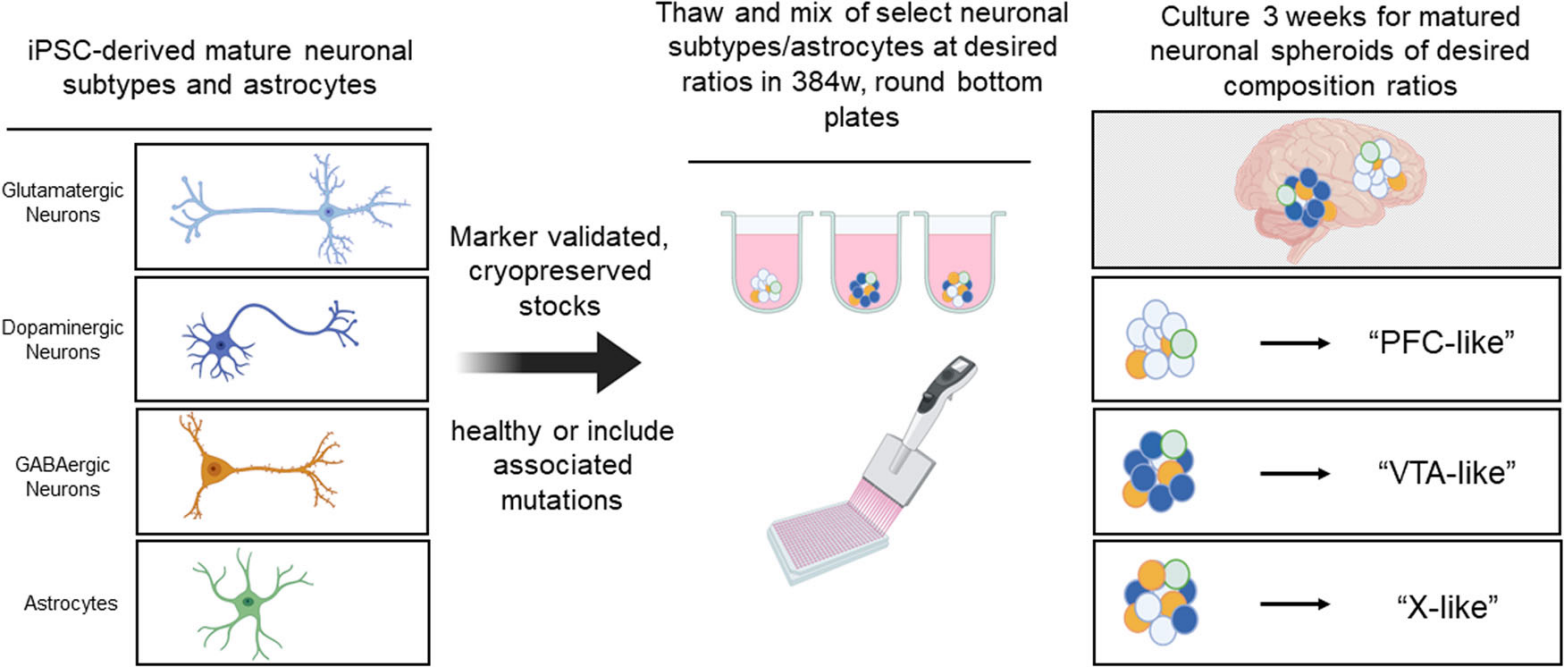
Schematic of brain region-specific neural spheroid generation protocol. Schematic showing method for generating brain region-specific neural spheroids; matured and differentiated iPSC-derived neural cells combined in pre-determined ratios reflecting the cell-type compositions of specific regions of the human brain. PFC-like prefrontal cortex like, VTA-like ventral tegmental like, X-like other brain region of choice. Schematic was created with Biorender.com.

**Fig. 2:**
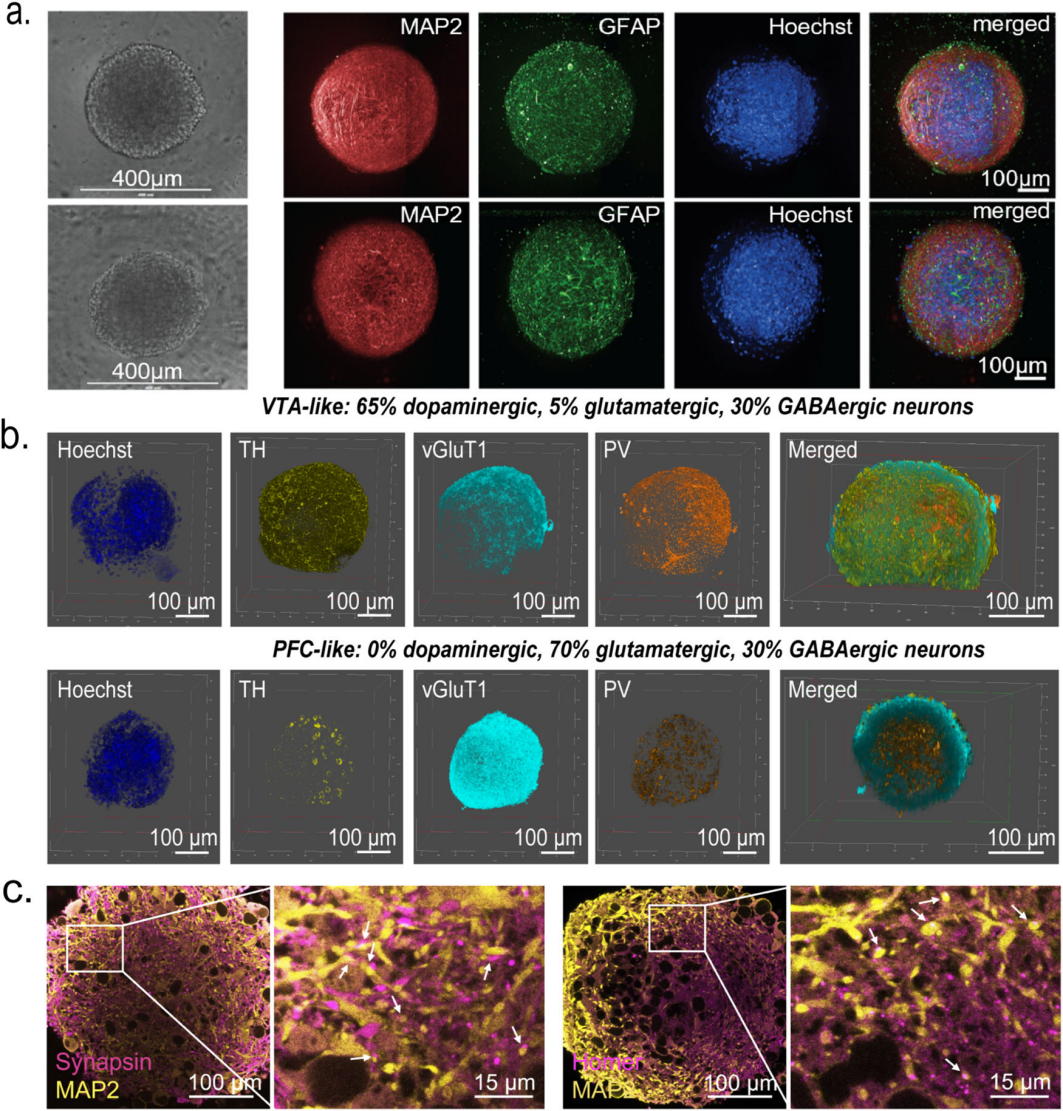
Immunofluorescence-based characterization of neural spheroids. **a** Spheroid types are similar in size regardless of cell-type composition (top panel contains mostly dopaminergic neurons, bottom contains mostly glutamatergic neurons; both spheroid types are 90% neuron 10% astrocyte); left to right: bright-field image, MAP2 neuronal marker, GFAP astrocyte marker, Hoechst nuclear stain, merged image of MAP2, GFAP, and Hoechst. 272 µm image stack acquired with 0.8 µm z-step, 20X water objective; represented as maximum projection image of 18 images every 20 slices (16 µm). Scale bar = 100 µm, except for bright field (scale bar = 400 µm). **b** 3D rendering from confocal z-stacks of VTA-like (top panel) and PFC-like (bottom panel) spheroids stained with neuronal cell-type markers; Left to right: Hoechst nuclear stain, tyrosine hydroxylase (TH, dopaminergic marker), vGluT1 (glutamatergic marker), and Parvalbumin (PV, GABAergic marker) along with a 3D rendering showing TH, vGluT, and PV expression in the center of each spheroid. Scale bar = 100 µm. **c** Confocal images showing MAP2 staining and presynaptic marker, synapsin (left panel), and postsynaptic marker, Homer (right panel), indicating functional synapses. Scale bar = 100 µm (full) or 15 µm (zoom-in).

We first sought to establish whether neurons and astrocytes differentiated from human-induced pluripotent stem cells (iPSC) could be aggregated in a scaffold-free environment of an ultra-low attachment (ULA) round bottom well into a co-culture spheroid system with functional neuronal activity. We mixed marker-validated, iPSC-derived glutamatergic, GABAergic, and dopaminergic neurons with astrocytes and seeded as cell mixtures of controlled ratios into 384-well, ULA round bottom plates to force cell aggregation into the formation of spheroids (Fig. 1). We observed spheroid formation after 3 days in culture in both prefrontal cortex (PFC)-like spheroids (70% glutamatergic, 30% GABAergic neurons) and ventral tegmental area (VTA)-like spheroids (65% dopaminergic, 5% glutamatergic, 30% GABAergic neurons), both consisting of 90% neurons and 10% astrocytes. The neuronal composition of the “VTA-like” and “PFC-like” spheroids was based on cell-type compositions from postmortem human brain studies^17–19^. Calcium activity was recorded after 21 days using a calcium fluorescence (Cal6) dye with a whole plate reader equipped with a high speed, high sensitivity EMCCD camera for fluorescent detection (the FLIPR Penta High-Throughput Cellular Screening System, “FLIPR”), suggesting formation of active, synchronized neuronal activity. Spheroids formed by this protocol were <400 μm in diameter after the maturation process (Fig. 2a) and had the homogenous spatial distribution of neurons and astrocytes (Fig. 2a and Supplementary Fig. S1). Furthermore, immunostaining for tyrosine hydroxylase (TH) showed higher positive cells in VTA-like spheroids compared to PFC-like spheroids (Fig. 2b and Supplementary Videos S1 (VTA-like) and  S2 (PFC-like)). Parvalbumin (PV) staining revealed similar compositions of GABAergic neurons between PFC- and VTA-like spheroids (Fig. 2b, Supplementary Fig S2b, and Supplementary Videos S1 and S2). We also detected higher vGluT1 mRNA and protein in PFC-like spheroids (Fig. 2b, Supplementary Fig. S2a, and Supplementary Video S2). The mature functional spheroids also expressed pre- and postsynaptic markers as shown by synapsin and homer staining, respectively, distributed evenly throughout spheroids, suggesting the presence of synaptic connections (Fig. 2c). As reference and for validation of the commercial, cryopreserved stocks of iPSC-derived neuronal cells used in this study, immunostaining for the presence of the different neuronal specific markers was also shown in 3-week 2D cultures, as shown in Supplementary Fig. S3.

**Fig. 3:**
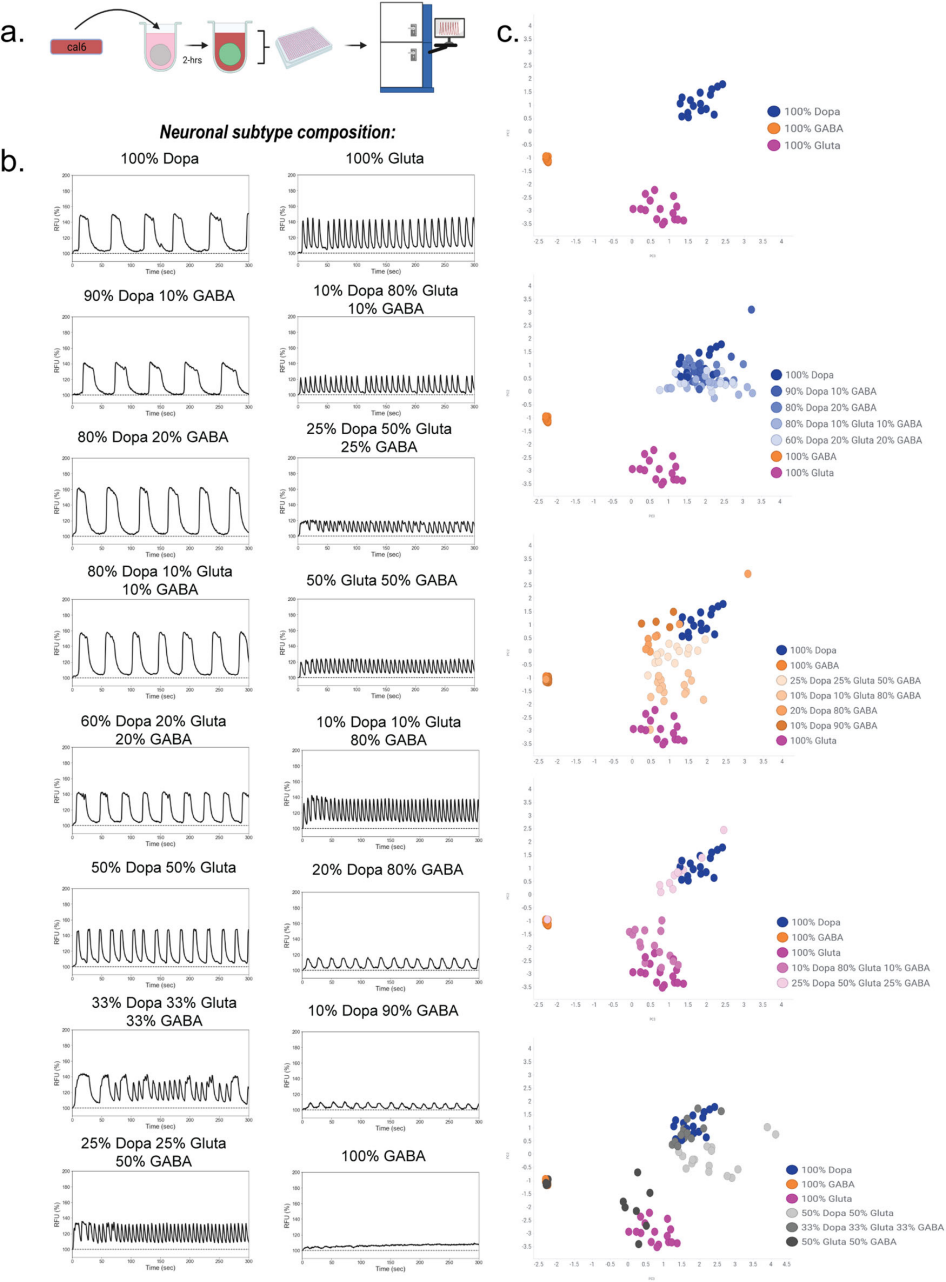
Calcium activity phenotypes are dependent on neuronal cell-type composition. **a** Schematic of experimental design; spheroids were incubated with Cal6 dye for 2-h prior to FLIPR recordings after a 3-week maintenance period. Schematic was created with Biorender.com. **b** Representative traces from 16 different spheroids that are 90% neuron and 10% astrocyte but differ by neuronal subtype composition. **c** Principal component analysis (PCA) was used as a dimension reduction algorithm to incorporate ten peak parameters extracted from the multiparametric peak analysis for all wells, and scatter plots were used to visualize the spatial distribution of calcium activity phenotypes for each spheroid type. Plots from top to bottom: (1) single-neuron spheroid (SNS) types (100% dopaminergic, glutamatergic, or GABAergic neurons). (2) Spheroids with majority dopaminergic neurons (shades of blue) relative to SNSs. (3) Spheroids with mostly GABAergic neurons (shades of orange) relative to SNSs. (4) Spheroids with mostly glutamatergic neurons (shades of pink) relative to SNSs. (5) Spheroids with equal distributions of neuronal cell types. Data were obtained from 16 technical replicates per spheroid type over one experiment. Raw data obtained from the multiparametric peak analysis was used for the PCA.

Calcium activity in the neural spheroids was recorded after 21 days using a calcium fluorescence (Cal6) dye with a whole plate reader equipped with a high speed, high sensitivity EMCCD camera for fluorescent detection (FLIPR® Penta High-Throughput Cellular Screening System, “FLIPR”), suggesting formation of active, synchronized neuronal activity (Fig. 3a). Calcium signal peak parameters were analyzed using ScreenWorks PeakPro 2.0 analysis and demonstrated the high well-to-well reproducibility of the assay. Specifically, we analyzed 17 peak parameters and selected 10 reproducible parameters with low variability using a previously described <30% coefficient of variance (%CV)) (Supplementary Table S1)^20^. To assess whether changing neuronal cell-type composition would impact phenotypic profiles, we performed a proof-of-concept study measuring calcium activity in 16 different spheroid types that contained 90% neurons and 10% astrocytes but differed in their neuronal subtype composition (Fig. 3b). Principal component analysis (PCA) was used to analyze the multiparametric peak data (Fig. 3c). We observed that spheroids including one neuronal type (single-neuron spheroids or SNSs, 90% neuron 10% astrocyte) consisting of only dopaminergic, glutamatergic, or GABAergic neurons form distinct clusters (Fig. 3c, topmost graph), suggesting neuronal-type specific phenotypic profiles, and spheroids with controlled gradient ratios of multiple neuronal cell types cluster near the SNS cluster with the dominant neuronal cell type (Fig. 3c). Together, these data show that altering neuronal cell-type composition within spheroids produces unique calcium activity phenotypes that reflect neuronal subtype ratios.

**Fig. 4:**
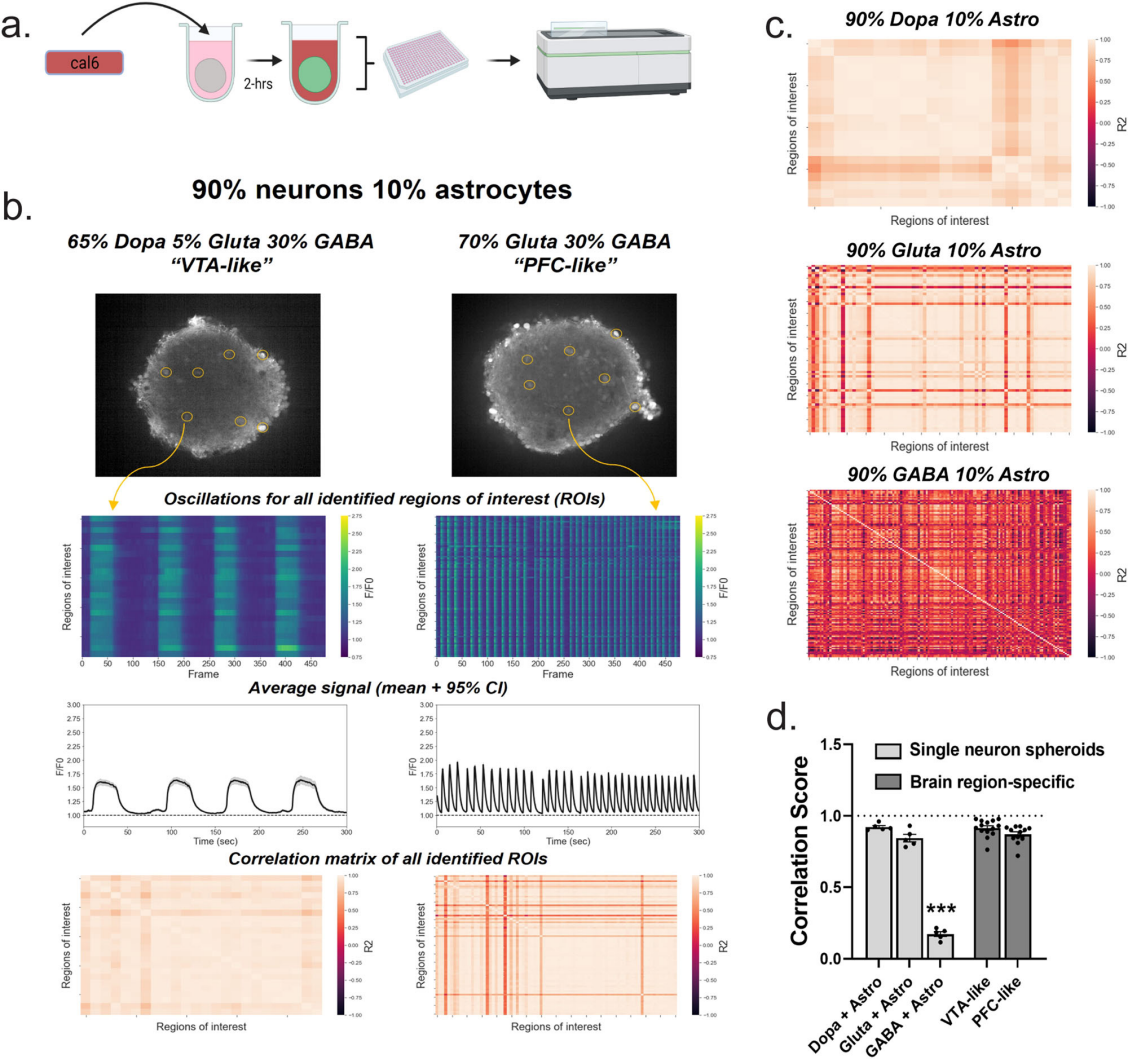
Synchronous calcium oscillations occur in brain region-specific spheroids as well as single-neuron spheroids with dopaminergic and glutamatergic, but not GABAergic, neurons. **a** Schematic of experimental design; spheroids were incubated in Cal6 dye for 2-hrs prior to confocal time series recordings after a 3-week maintenance period. Schematic was created with Biorender.com. **b** Calcium activity in brain region-specific spheroids modeling the ventral tegmental area (VTA-like) and prefrontal cortex (PFC-like); Top: visual of the automated region of interest (ROI) detection used to examine calcium activity within several cells in each spheroid; Second from top: calcium activity from all identified ROIs shown in the form of a heatmap, with tick marks for ROIs in increments of 5 on the left *y* axis, dF/F0 on the right *y* axis, and video frame on the *x* axis; Second from bottom: Average signal of all identified ROIs plus 95% confidence interval; Bottom: Correlation matrices displaying *R*^2^ values as a heatmap for all identified ROIs within a spheroid. Regions of interest (ROIs) with oscillatory patterns were automatically identified using the LC_Pro plugin through ImageJ and the activity of all identified ROIs was plotted as a heatmap and population activity was represented by the mean plus 95% confidence interval of all identified ROIs (time series trace). **c** Correlation matrices from representative single-neuron spheroids showing synchronicity values of all identified ROIs. **d** Quantification of synchrony through a correlation score, obtained through the mean *R*^2^ value for each spheroids’ correlation matrix. Data were run in technical replicates of *n* = 4–6 per plate across one experiment for SNSs and three separate experiments for brain region-specific spheroids. **d** One-way ANOVA (*F*_(4,35)=_100.7, *P* < 0.0001) was followed up with Dunnett’s posthoc (*P* < 0.0001 for all groups compared to GABA + Astro spheroids), data is represented as mean ± SEM, ***P < 0.001.

After establishing culture maturation conditions and maintenance of single and multiple neuronal subtypes, we next characterized PFC- and VTA-like spheroids (described in Fig. 2) by confocal microscopy to measure calcium activity from individual cells, rather than population level (as measured by FLIPR), within either the single-neuron spheroids (SNSs) or brain region-specific spheroids (schematic, Fig. 4a and Supplementary Videos S3 and S4). Correlation matrices, which are used to show correlation coefficients between variables, generated from individual cell calcium activity between identified regions of interest (ROIs) were plotted as heatmaps, and the average correlation coefficient (R^2^) value from each spheroid was calculated as correlation scores (Fig. 4b, c), which were used as a measure of neuronal activity synchronicity. VTA- and PFC-like spheroids displayed unique phenotypic profiles like phenotypes displayed by SNSs with the corresponding dominant neuronal cell types (Fig. 4b and Supplementary Fig. S5). In addition, robust synchronous activity was observed in SNSs with dopaminergic and glutamatergic neurons along with both brain region-specific spheroid types, but not in SNSs with GABAergic neurons (Fig. 4c, d and Supplementary Videos S5 and S6). While astrocytes were not necessary for activity, astrocyte presence altered peak parameters in SNSs containing dopaminergic or glutamatergic neurons (Supplementary Fig. S5). Given the role astrocytes play in neurotransmitter release, synaptic plasticity and neurological diseases^21–23^, spheroids used throughout the rest of the study were all made to include 10% astrocytes (Supplementary Fig. S5). Together, these data indicate that brain region-specific spheroids display unique phenotypic profiles depending on the cell type, and that changes in synchronous neuronal activity in these spheroids may occur through GABAergic mechanisms.

### Phenotypic profiles in brain region-specific spheroids can be differentially modulated with compounds targeting neuronal subtype receptors

We next validated spheroid functional response via treatment with reference compounds that target receptors on each neuronal subtype (Supplementary Figs. S6 and S7 and Supplementary Table S2). In both spheroid types, VTA- and PFC-like, treatment with the GABA_A_ receptor (GABA_A_R) agonist muscimol led to complete inhibition of activity (Supplementary Figs. S6 and S7 and Supplementary Table S2). Conversely, treatment with GABA_A_R antagonist, bicuculline, induced spheroid type-dependent changes: in VTA-like spheroids, enhanced activity was apparent through increases in peak count, rate, and amplitude with decreases in peak rise and decay time, while in PFC-like spheroids, peak count was reduced while peak amplitude and peak width were increased, suggesting different functional responses between the spheroids to GABA_A_R antagonism (Supplementary Fig. S6 and Supplementary Table S2). To target glutamatergic neurons, spheroids were treated with the α-amino-hydroxy-5-methyl-4-isoxazolepropionic acid receptor (AMPAR) antagonist, CNQX, which inhibited all activity in PFC-like spheroids but led to increases in peak rise time and peak count in VTA-like spheroids (Supplementary Fig. S6 and Supplementary Table S2). The N-methyl-d-aspartate receptor (NMDAR) antagonist, memantine, increased peak count and rate and decreased amplitude in both spheroid types (Supplementary Fig. S6 and Supplementary Table S2). SCH23390 was used to block stimulatory G-protein coupled dopamine 1/5 receptors (D1Rs) while sulpiride was used to block inhibitory G-protein coupled dopamine 2/3 receptors (D2Rs). In both spheroid types, D1R antagonism with SCH23390 led to total inhibition in both spheroid types while D2R antagonism with sulpiride increased peak count in PFC-like spheroids and peak width in VTA-like spheroids (Supplementary Fig. S6 and Supplementary Table S2). Together, these data show predictable pharmacological functional responses occurred in brain region-specific spheroids based on their neuronal cell-type composition.

### Incorporating genetically engineered GABAergic neurons expressing APOE e4/4 allele produces a predictive calcium activity phenotype that is reversed with clinically approved treatments for Alzheimer’s disease

To develop a neural spheroid Alzheimer’s disease (AD) drug screening platform, we used GABAergic neurons that were genetically engineered to carry the apolipoprotein e4/4 (APOE4) allele, a genotype known to increase the risk for AD^24,25^. PFC-like spheroids were made either containing 30% APOE3 (Wt) or APOE4 (AD-associated allele) GABAergic neurons, with SNSs with Wt APOE3 or APOE4 GABAergic neurons as controls for GABAergic neuron activity and viability. After a 3-week maturation period, there was no significant differences in spheroid viability or nuclei count between APOE3 and APOE4 GABAergic neurons, suggesting that functional differences observed between genotypes are not because of cell viability differences caused by the APOE4 allele (Supplementary Fig. S8).

**Fig. 5:**
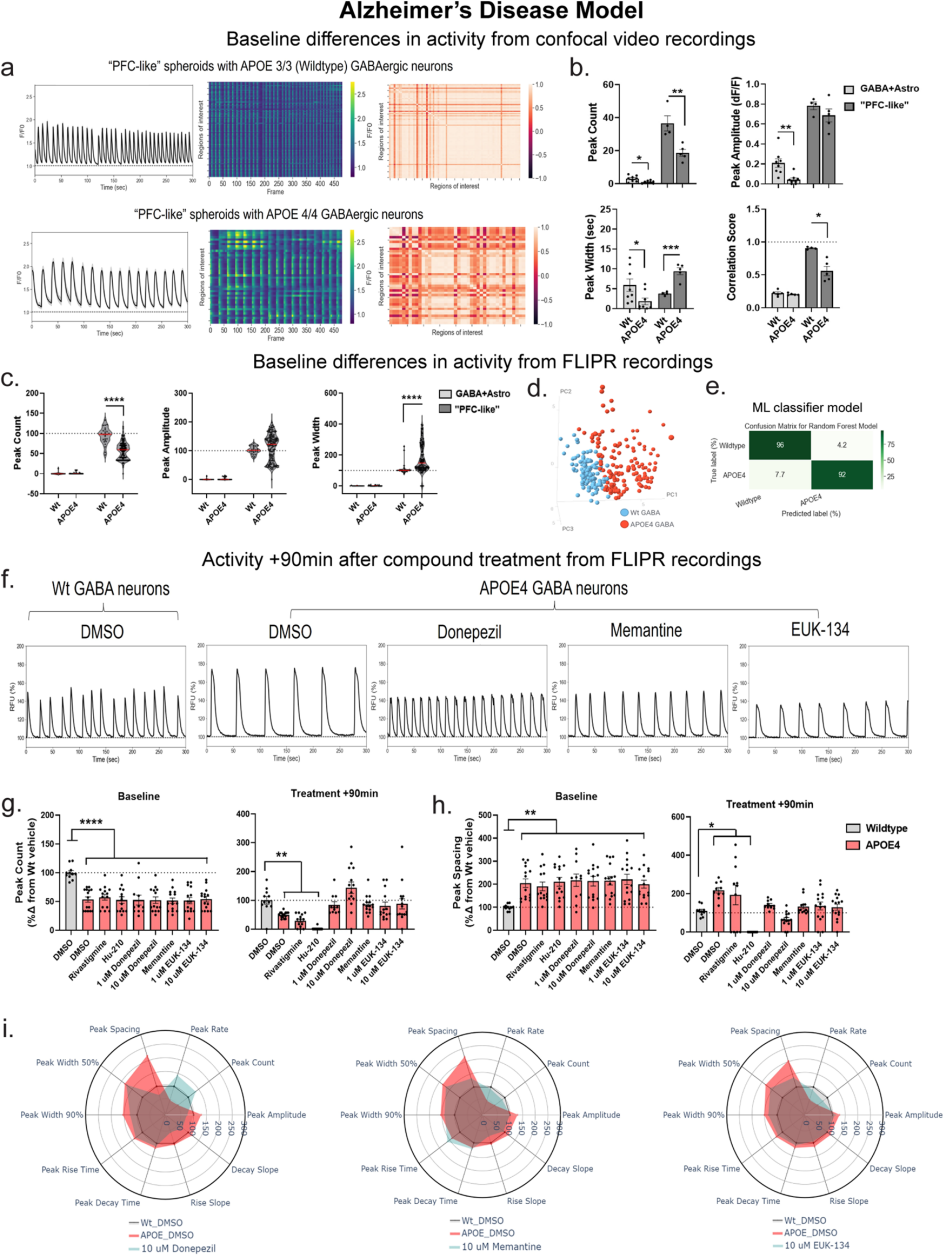
Clinically approved compounds to treat symptoms of Alzheimer’s disease (AD) reverse deficits caused by the incorporation of APOE4/4 GABA neurons in PFC-like spheroids. **a**, **b** Data collected from confocal recordings**. a** Baseline phenotypic differences in PFC-like spheroids containing GABA neurons expressing APOE3/3 (wild type, top panel) or APOE4/4 (bottom panel), a gene variant associated with AD; left: average signal across all identified ROIs, middle: heatmap showing activity of all identified ROIs, Right: correlation matrix showing synchronicity measurements of ROIs. **b** Quantification of peak count (SNS: *t*_(14)_ = 2.92, *P* = 0.011; PFC-like: *t*_(7)_ = 3.81, *P* = 0.007), amplitude (SNS: *t*_(14)_ = 3.58, *P* = 0.011), width (SNS: *t*_(14)_ = 2.27, *P* = 0.039; PFC-like: *t*_(7)_ = 5.46, *P* = 0.0009), and synchronicity from confocal recordings (PFC-like: *t*_(7)_ = 4.36, *P* = 0.003). **c**–**i** Data collected from FLIPR recordings. **c** Baseline peak count (SNS: *P* = 0.25; PFC-like: *P* < 0.0001), amplitude (SNS: *P* = 0.21; PFC-like: *P* = 0.056), and width (SNS: *P* = 0.18; PFC-like: *P* < 0.0001) from all wells recorded with FLIPR. **d** PCA on baseline multiparametric peak data represented as a scatterplot displaying individual values. **e** Predictive labeling of genotype based on PCA data is 94% accurate using the Random Forest machine learning classifier model; Data represented as a confusion matrix showing accurate and erroneous error labels for each genotype, values represented as percentages. **f** Representative time series traces 90 min after Wt and APOE4 spheroids were treated with compounds used to treat AD. **g**, **h** Peak count (**g**) and peak spacing (**h**) in Wt PFC-like spheroids treated with DMSO and APOE4 spheroids divided by treatment group at baseline (left) and 90 min after treatment (right); Donepezil, Memantine, and EUK-134 restore deficits in peak count (peak count, baseline: *F*_(8,115)_ = 7.05; *P* < 0.0001; treatment: *F*_(8,114)_ = 12.4, *P* < 0.0001; peak spacing, baseline: *F*_(8,115)_ = 3.06, *P* = 0.004; treatment: *F*_(8,102)_ = 10.22, *P* < 0.0001). **i** Radar plots showing phenotypic data across ten peak parameters measured 90 min after treatment with DMSO in Wt (gray) and APOE4 (red) spheroids, along with treatment with either Donepezil, Memantine, and EUK-134 (teal). For confocal recordings, *n* = 4–8 per group over two separate experiments; For FLIPR recordings, *n* = 249 samples over three separate experiments. Data from (**b**, **g**, **h**) represented as mean ± SEM, **b** analyzed with unpaired *t* tests for each spheroid type and (**g**, **h**) analyzed with one-way ANOVA followed up with Dunnett’s posthoc. Data from (**c**) represented as violin plots with median indicated by red line; Mann–Whitney unpaired *t* test used to compare medians between groups, (**i**) represented as radar plots showing group averages for each peak parameter analyzed. *P < 0.05, **P < 0.01, ***P < 0.001, ****P < 0.0001).

Both PFC-like and SNSs with APOE4 GABAergic neurons displayed reduced peak count by ROI analysis (Fig. 5a, b). However, peak amplitude was reduced only in SNSs with APOE4 GABAergic neurons while peak width was decreased in SNSs but increased in PFC-like spheroids with APOE4 GABAergic neurons (Fig. 5a, b). In PFC-like spheroids, the incorporation of APOE4 GABAergic neurons also disrupted synchronous neuronal activity, as indicated by the reduction in correlation scores (Fig. 5a, b and Supplementary Videos S7 and S8). FLIPR recordings of baseline activity replicated the confocal recording, showing that APOE4 GABAergic neurons in PFC-like spheroids caused a reduction in peak count and an increase in peak width (Fig. 5c). Given the low synchronicity of SNSs with GABAergic neurons, the FLIPR was unable to detect peaks in these spheroids (Fig. 5c). PCA on baseline multiparametric peak data represented as a scatterplot displaying individual values was able to segregate Wt (APOE3) PFC-like spheroids and APOE4 PFC-like spheroids (Fig. 5d). We also implemented a random forest classifier (RFC) supervised machine learning algorithm, described in methods, to assess prediction accuracy of genotypes based on baseline FLIPR data in PFC-like spheroids to quantify predictivity of the APOE4 phenotype. The RFC model accurately predicted the label of 96% of Wt (APOE3) PFC-like spheroids and 92% of APOE4 PFC-like spheroids (Fig. 5e). These data show that APOE4 GABAergic neurons produce deficits in baseline calcium activity that are highly predictive when tested against the RFC machine learning algorithm.

We next sought to investigate whether APOE4-induced functional deficits in PFC-like spheroids could be reversed following treatment with three clinically approved compounds used to treat the symptoms of AD in humans along with two preclinical compounds being developed for AD. The clinically approved compounds included cholinesterase inhibitors (Rivastigmine and Donepezil) along with an NMDAR antagonist (Memantine) while the preclinical compounds were Hu-210, a cannabinoid agonist, and EUK-134, which prevent oxidative stress^26,27^. FLIPR recordings were obtained 90-min after compound treatment, in the same spheroids analyzed in Fig. 5c–e. All data were normalized to Wt dimethyl sulfoxide (DMSO)-treated controls. Baseline deficits were consistent between all treatment groups prior to compound addition and show significant reductions in peak count and increases in peak spacing among all treatment groups in APOE4 PFC-like spheroids compared to Wt controls (Fig. 5g, h). Three compounds were found to reverse deficits caused by APOE4 GABAergic neurons including Memantine (10 µM), Donepezil (1 and 10 µM), and EUK-134 (1 and 10 µM) since deficits in peak count and spacing were no longer significantly different from Wt DMSO-treated spheroids (Fig. 5f–h, panels labeled Treatment +90 min). In Wt PFC-like spheroids, these same compounds also increased peak count and decreased peak spacing, which may indicate a general rather than APOE4-specific mechanism (Supplementary Fig. S9). Radar plots for each of these three compounds illustrate the phenotypic profiles across all peak parameters 90-min after treatment compared to DMSO-treated controls (Fig. 5i). These findings show that functional deficits presented in the APOE4 GABAergic PFC-like neural spheroids can be partially reversed by select compounds used to treat AD symptoms.

### Naloxone reverses deficits induced by chronic DAMGO treatment in PFC-like but not VTA-like spheroids

**Fig. 6:**
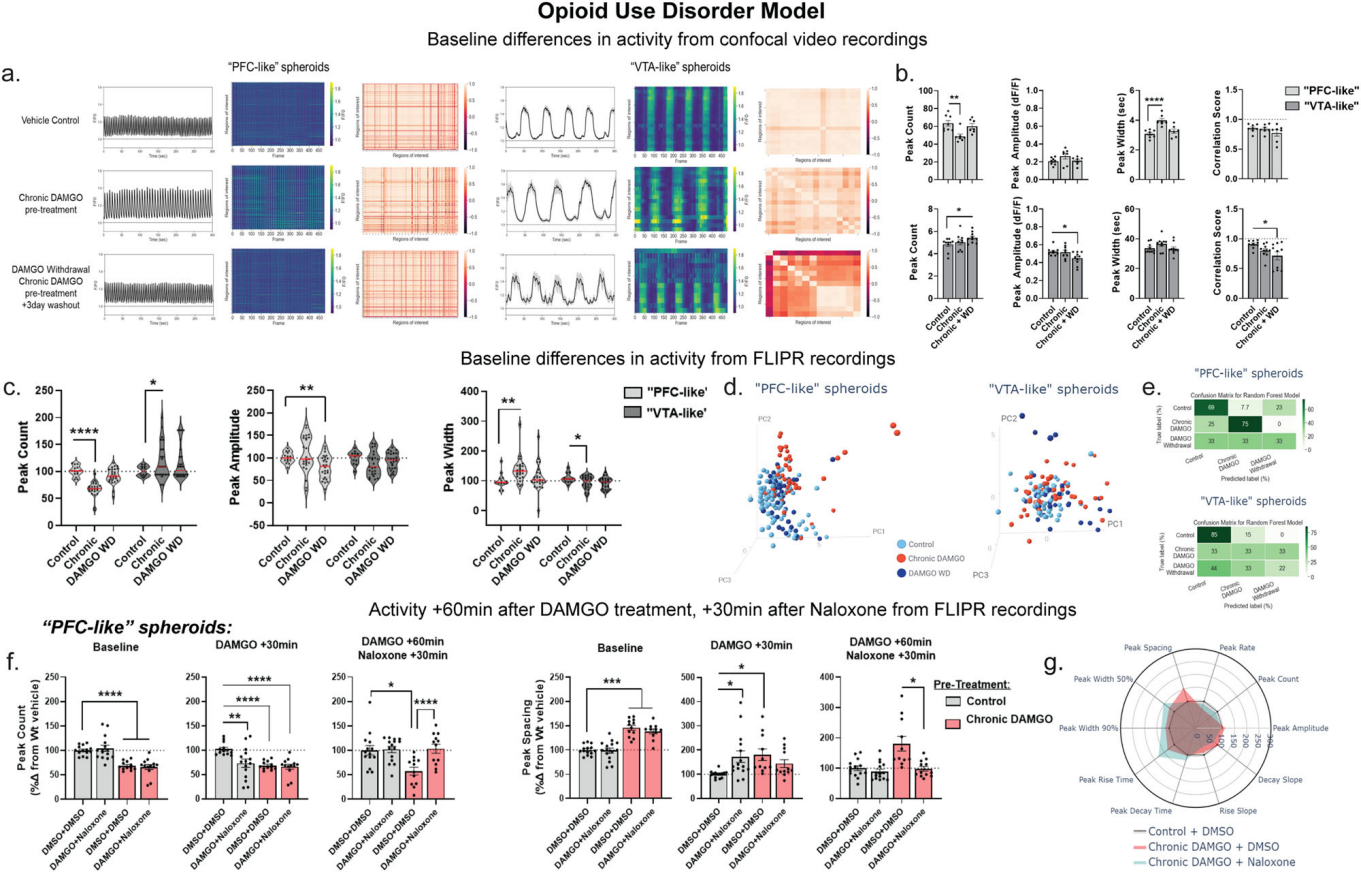
Naloxone (MOR antagonist) reverses deficits induced by chronic opioid treatment in PFC-like but not VTA-like spheroids in spheroids modeling opioid use disorder. **a**, **b** Data collected from confocal recordings. **a** Baseline phenotypic differences in PFC- and VTA-like spheroids pre-treated with either vehicle (water, top panel), chronic DAMGO (middle panel), or chronic DAMGO plus a 3-day washout to model drug withdrawal**;** left: average signal across all identified ROIs, middle: heatmap showing activity of all identified ROIs, right: correlation matrix showing synchronicity measurements of ROIs. **b** Quantification of peak count (PFC-like: *F*_(2,19)_ = 7.17, *P* = 0.005; VTA-like: *F*_(2,26)_ = 2.26, *P* = 0.097), amplitude (PFC-like: *F*_(2,19)_ = 1.99, *P* = 0.16; VTA-like: *F*_(2,26)_ = 4.03, *P* = 0.03), width (PFC-like: *F*_(2,19)_ = 14.91, *P* = 0.0001; VTA-like: *F*_(2,26)_ = 2.04, *P* = 0.15), and synchronicity (PFC-like: *F*_(2,19)_ = 0.74, *P* = 0.28; VTA-like: *F*_(2,26)_ = 4.27, *P* = 0.025) from confocal recordings. **c**–**f** Data collected from FLIPR recordings. **c** Quantification of baseline peak count, amplitude, and width from all wells recorded with FLIPR (peak count: PFC-like: *F*_(2,61.7)_ = 36.9, *P* < 0.0001, VTA-like: *F*_(2,53.5)_ = 2.04, *P* = 0.14; amplitude: PFC-like: *F*_(2,47.18)_ = 7.23, *P* = 0.002, VTA-like: *F*_(2,54.4)_ = 2, *P* = 0.15; width: PFC-like: *F*_(2,40.3)_ = 5.36, *P* = 0.007 VTA-like: *F*_(2,53.5)_ = 3.33, *P* = 0.04). **d** PCA on baseline multiparametric peak data represented as a scatterplot displaying individual values in PFC-like (left) and VTA-like spheroids (right). **e** Predictive labeling of pre-treatment group based on PCA data using Random Forest machine learning classifier model. Data represented as a confusion matrix showing accurate and erroneous error labels for each pre-treatment group, values represented as percentages. **f** Peak count and peak spacing in PFC-like spheroids. Data show baseline differences between spheroids with vehicle vs chronic DAMGO pre-treatment (left panel), 30 min after DAMGO treatment (middle panel), and 60 min after DAMGO treatment plus 30 min after naloxone treatment. Data show that naloxone is able to reverse deficits induced by chronic DAMGO pre-treatment and DAMGO treatment. **g** Radar plots showing phenotypic data across 10 peak parameters measured 60 min after treatments with either DMSO, DAMGO, and/or naloxone. For confocal recordings, *n* = 7–8 per group over two separate experiments; for FLIPR recordings, *n* = 171 (PFC-like) and 135 samples (VTA-like) over three separate experiments. Data from (**b**, **f**) represented as mean ± SEM, analyzed with one-way ANOVA followed up with Dunnett’s posthoc. Data from (**c**) represented as violin plots with median indicated by red line; Brown–Forsythe and Welch. One-way ANOVA used to compare medians between groups, **i** represented as radar plots showing group averages for each peak parameter analyzed. *P < 0.05, **P < 0.01, ***P < 0.001, ****P < 0.0001).

We next modeled opioid use disorder (OUD) by developing a protocol intended to investigate various facets of addiction including drug intake and withdrawal^6^. We modeled drug intake by chronically treating spheroids with 10 µM DAMGO, an MOR agonist, every other day for 10 days. DAMGO withdrawal was modeled in the same way, but spheroids were not treated on the final day such that this treatment group received control media 3 days prior to recording (Fig. 6a). DAMGO pre-treatment did not impact spheroid viability (Supplementary Fig. S8c).

Chronic DAMGO treatment decreased peak count and increased peak width in PFC-like spheroids, while DAMGO washout spheroids modeling withdrawal did not have a significant change from control spheroids in peak count, amplitude, or width (Fig. 6a, b). In VTA-like spheroids, chronic DAMGO pre-treatment did not change baseline activity but DAMGO withdrawal significantly increased peak count, decreased peak amplitude, and disrupted synchrony compared to control spheroids (Fig. 6a, b). We observed similar differences using the FLIPR as were observed with the automated confocal recordings (Fig. 6c and Supplementary Fig. S10). PCA found 69% accurate prediction of phenotypes in chronic DAMGO PFC-like spheroids but only 33% accuracy with the DAMGO withdrawal spheroids (Fig. 6d). For VTA-like spheroids, the RFC model accurately predicted 85% of control spheroids but only accurately identified 33% and 22% of chronic DAMGO-treated or DAMGO withdrawal spheroids, respectively (Fig. 6e), suggesting the phenotypic differences are not predictive in a classifier model.

Spheroids were then treated with 10 µM DAMGO, and calcium activity recorded 30-min later, followed immediately by 10 µM naloxone, a MOR antagonist used clinically to reverse opioid overdoses. Control and chronic DAMGO-treated spheroids were either treated with DMSO prior to each recording (DMSO + DMSO) or DAMGO followed by naloxone (DAMGO + Naloxone). Thirty minutes after treatment with either DMSO or DAMGO, acute DAMGO significantly reduced peak count in control spheroids and chronic DAMGO pre-treated spheroids continued showing lower peak count (Fig. 6f, DAMGO + 30 min). For peak spacing, acute DAMGO treatment in control spheroids increased peak spacing, and peak spacing remained significantly increased in chronic DAMGO-treated spheroids that were treated with DMSO, but not DAMGO (Fig. 6f, DAMGO + 30 min). Spheroids were then treated with either DMSO or naloxone and recorded 30 min later. We observed naloxone reversed deficits in peak count and spacing in both control spheroids acutely treated with DAMGO as well as chronic DAMGO pre-treated spheroids (Fig. 6f, g, DAMGO + 30 min, Naloxone +60 min). These findings show that DAMGO treatment causes deficits in peak count and spacing that can be reversed by blocking MORs in PFC- but not VTA-like spheroids (Fig. 6e).

### Functional assembloids made from conjoining VTA- and PFC-like spheroids can be used to model neural circuitry

**Fig. 7:**
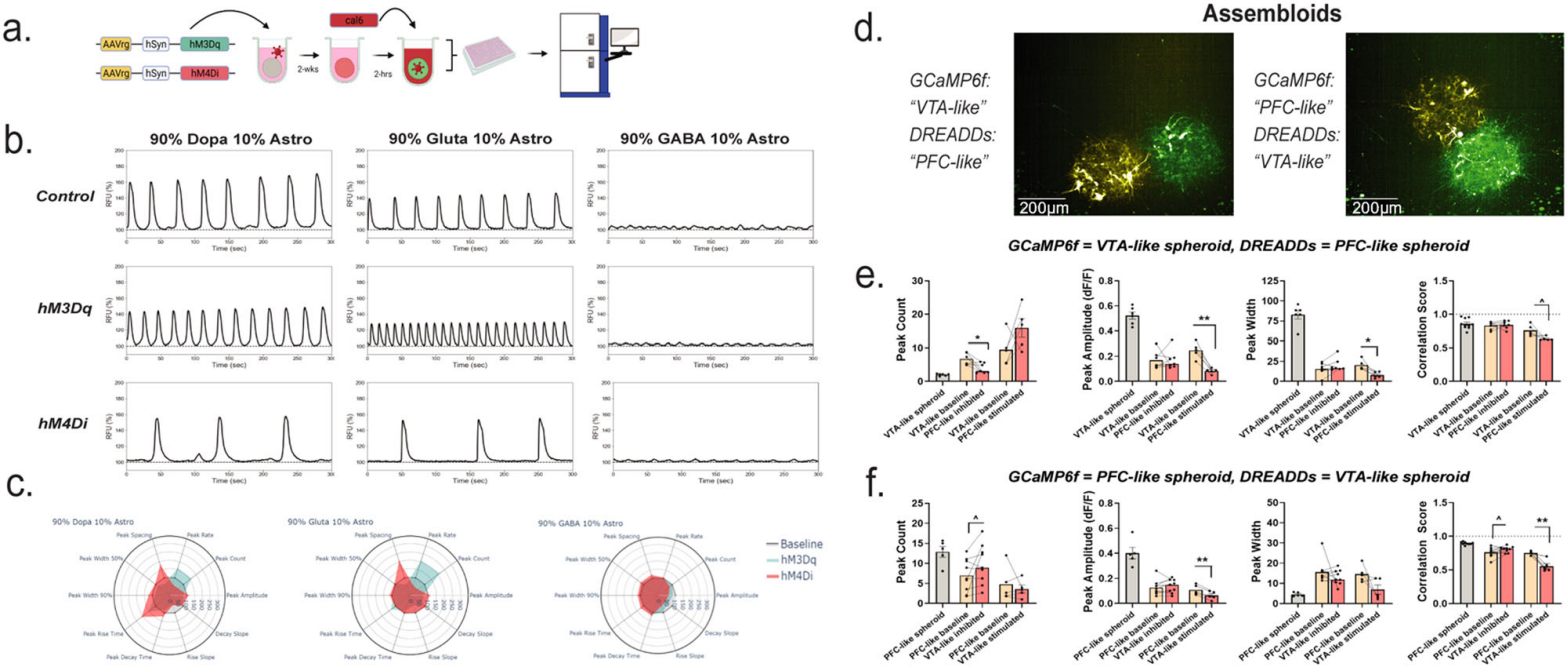
Functional assembloids can be made from conjoined spheroids to model neural circuitry. **a** Schematic showing that spheroids can be transfected with DREADDs viruses tagged with mCherry and activity can be recorded with a FLIPR at 3 weeks using a cal6 dye. Schematic was created with Biorender.com. **b** Activity from single-neuron spheroids (SNSs) comprised of 90% neuron and 10% astrocytes can be recorded and displays similar phenotypes in spheroids transfected with stimulatory (hM3Dq) or inhibitory (hM4Di) DREADDs viruses. Top panel: vehicle control-treated spheroids expressing no DREADDs virus; middle panel: 60-min after treatment with CNO to activate DREADDs virus and induce stimulatory activity; bottom panel: 60-min after treatment with CNO to activate DREADDs virus and induce inhibitory activity. **c** Radar plots showing multiparametric peak alterations across 10 peak parameters for both stimulatory (teal) and inhibitory (red) DREADDs in SNSs with dopaminergic, glutamatergic, and GABAergic neurons, respectively. **d**–**f** is from confocal recordings. **d** Representative images of two assembloids where one component expresses GCaMP6f and the other expresses either stimulatory (hM3Dq) or inhibitory (hM4Di) DREADDs viruses; Left: VTA-like expressing GCaMP6f, PFC-like expressing hM4Di. Right: PFC-like expressing GCaMP6f, VTA-like expressing hM4Di. Scale bar = 200 µm. **e**, **f** Quantification of baseline vs 90-min activity for peak count (hM4Di: *t*_(6)_ = 3.15, *P* = 0.019; hM3Dq: *t*_(4)_ = 1.6, *P* = 0.19), amplitude (hM4Di: *t*_(6)_ = 0.04, *P* = 0.97; hM3Dq: *t*_(4)_ = 7.6, *P* = 0.002), width (hM4Di: *t*_(6)_ = 1.93, *P* = 0.1; hM3Dq: *t*_(4)_ = 3.29, *P* = 0.03), and synchronicity (hM4Di: *t*_(6)_ = 0.35, *P* = 0.74; hM3Dq: *t*_(4)_ = 2.7, *P* = 0.05) of the VTA-like component (**e**) or PFC-like (**f**) component (peak count: hM4Di: *t*_(8)_ = 1.94, *P* = 0.09, hM3Dq: *t*_(4)_ = 0.65, *P* = 0.55; amplitude: hM4Di: *t*_(8)_ = 0.8, *P* = 0.45, hM3Dq: *t*_(4)_ = 7.3, *P* = 0.002; width: hM4Di: *t*_(8)_ = 1.003, *P* = 0.35, hM3Dq: *t*_(4)_ = 2.11, *P* = 0.103; synchronicity: hM4Di: *t*_(8)_ = 2.28, *P* = 0.052, hM3Dq: *t*_(4)_ = 4.95, *P* = 0.008) of assembloids before (yellow) and after (red) CNO was added to media to activate inhibitory (hM4Di) or stimulatory (hM3Dq) DREADDs in the PFC-like (**e**) or VTA-like (**f**) component of assembloids. **b**, **c** *n* = 8–12 per group over three separate experiments; **d**–**f** *n* = 4–9 per group over two experiments. Data from (**c**) are represented as mean and from (**e**, **f**) are represented as mean plus individual values before and after CNO was used to activate DREADDs. Data from (**e**, **f**) were analyzed with paired *t* tests *P < 0.05, **P < 0.01, ***P < 0.001, ****P < 0.0001.

To extend this technology to model neural circuitry, we generated assembloids with the VTA-like and PFC-like spheroids. To activate the activity of neural circuits in assembloids, we first tested whether calcium activity could be manipulated using a chemogenetic approach (Fig. 7a). We transduced spheroids with retrograde AAV viruses inserting either stimulatory (hM3Dq) or inhibitory (hM4Di) designer receptors exclusively activated by designer drugs (DREADDs). Clozapine-N-oxide (CNO) was used at a 1 µM concentration to activate the DREADDs receptors, and representative time series traces as well as radar plots show the effect 30-min after treatment with FLIPR recordings (Fig. 7b). For dopaminergic and glutamatergic SNSs, chemogenetic activation increased peak count and rate while reducing parameters such as peak spacing and width, while the opposite was observed for these spheroids when subjected to chemogenetic inhibition (Fig. 7b, c). We further showed that spheroids can express the genetically encoded calcium indicator, GCaMP6f, and that phenotypes within single VTA-like and PFC-like spheroids resemble what we observed from spheroids incubating in Cal6 dye (Supplementary Fig. S11a, b).

We modeled neural circuit-specific projections by pairing a PFC- and VTA-like spheroid together such that one expressed GCaMP6f and the other expressed either the inhibitory DREADDs virus, hM4Di, or the stimulatory DREADDS virus, hM3Dq (Fig. 7d). We first recorded assembloids where the VTA-like component of the assembloid expressed GCaMP6f and the PFC-like component expressed hM4Di or hM3Dq and recorded both baseline and 60-min after CNO activity (Fig. 7d, left image, e and Supplementary Fig. S11c). Inhibiting the PFC-like component of the assembloid significantly reduced peak count to the level of a single VTA-like spheroid (Fig. 7e and Supplementary Fig. S11c). Conversely, stimulating the PFC-like component increased peak count from baseline, though this was not statistically significant (Fig. 7e and Supplementary Fig. S11e). Furthermore, baseline peak amplitude and width, along with synchronicity were not changed after inhibiting the PFC-like component of the assembloid, but a significant reduction across these three parameters was observed after stimulating the PFC-like component (Fig. 7e and Supplementary Fig. S11c, e).

We then recorded from assembloids where the PFC-like component expressed GCaMP6f and the VTA-like component expressed hM4Di or hM3Dq (Fig. 7f and Supplementary Fig. S11d). Inhibiting the VTA-like component of the assembloid significantly increased peak count from baseline, though this was not observed when stimulating the VTA-like component of the assembloid (Fig. 7f and Supplementary Fig. S11d, f). Conversely, peak amplitude was not affected after inhibiting the VTA-like component of the assembloid but was significantly reduced after stimulating the VTA-like component, while peak width was unaffected by both stimulation or inhibition (Fig. 7f and Supplementary Fig. S11d, f). Lastly, we observed synchronicity was increased after inhibiting the VTA-like component of the assembloid, though this was only a statistical trend toward significance, but significantly reduced after stimulating the VTA-like component (Fig. 7f and Supplementary Fig. S11d, f). Taken together, these data show that brain region-specific neural circuits can be modeled with these spheroids, and that calcium activity phenotypes can be modulated by manipulating different components of the assembloid.

## Discussion

While organoid models have made significant inroads as 3D neural models to investigate brain development and diseases, their complexity hinders their use as robust high-throughput drug screening (HTS) assay platforms, including batch-to-batch variation in both size and cell composition heterogeneity, limited differentiation of neuronal cell types, and lengthy differentiation and maturation times^9,28,29^. Spheroids have emerged as physiologically relevant functional neural 3D tissue models with cell-type complexity, albeit without the tissue-like anatomy, and with the robustness and reproducibility necessary for HTS. Available neural spheroid models have been generated by differentiating neural stem cells into neurons and glial subtypes but these are limited to modeling cortical brain regions^12,14,30^. We used a different approach to making neural spheroids by developing cell aggregation, scaffold-free protocols with iPSC-differentiated neural cell types, in ratios that reflected those in different regions of the human brain. The protocol produced spheroids homogenous in size and cell distribution, without a necrotic core, and with a final neuronal-type composition similar to that initially seeded. Spheroids were active as shown through synaptogenesis and distinct spontaneous intracellular calcium oscillation patterns depending on cell-type composition after a 3-week maturation period. It is also worth noting that we observed phenotypic profiles between single-neuron spheroids that were more distinctly unique when they contained astrocytes. Given the vital role astrocytes play in synaptic connections and neurological disease^21,22,31^, we generated all neural spheroids with a ratio of 90% neurons and 10% astrocytes to improve the physiological relevance. Furthermore, bulk generation of spheroids is easily adapted for standard Aggrewell microwell plates, instead of 384-well plates that were used in this study, and half-media changes are readily amenable to using spheroids compatible liquid exchange platforms such as the MultiFlo FX (Biotek). Together, this protocol produces neural spheroids with high reproducibility, improved translatability, relatively short maturation period, and HTS compatibility.

In our study, we have shown that calcium activity can be measured using either a calcium dye (Cal6) or genetically encoded calcium indicator (GCaMP6f) as a primary readout for neural spheroid activity under healthy and diseased conditions. We recorded both single-cell recordings with an automated confocal microscope and population, well-based calcium fluorescence measurements with a fluorescent imaging plate reader (FLIPR). We used a multiparametric analysis to quantitate calcium activity peak features and measure differences between spheroids of different composition or with disease mutations. We applied a machine learning approach to quantify the predictability of calcium activity phenotypes under healthy or diseased conditions by using a random forest classifier model and were able to demonstrate these spheroids as a reproducible endpoint to establish the effects of drugs on correcting two different disease phenotypes. While the population-based readout with FLIPR limited the detection of activity in spheroids with high GABAergic neurons, the single-cell image-based analysis was able to quantitate synchronicity of the neural network, which became an important additional parameter to distinguish activity in spheroids of different composition and disease mutations, as well as corrective effects of drug treatments.

While calcium oscillation are not fully understood in vivo, data supports they are a result of complex synaptic formations within neuronal networks and adaptation to persistent neural inputs, both excitatory and inhibitory^32^. Our proof-of-concept experiment (Fig. 3) evaluated calcium oscillations of spheroids with 16 different cell-type compositions, and we observed distinct peak phenotypes when the neuronal population is dominated by dopaminergic neurons compare to glutamatergic neurons. The increased peak width associated high dopaminergic population might be a result of distinct firing characteristics and kinetics of dopaminergic neurons, as it was previously established that dopaminergic neurons exhibit phasic firing that is associated with robust GCaMP6 activity, and that dopaminergic neurons exhibit broad waveforms and slow firing rates^33–35^. Furthermore, increased ratio of GABAergic neurons is found to be associated with lower peak amplitude, which could be a result of increased negative feedback^32^. Simultaneous electrophysiology recording and the incorporation of genetically encoded transmitter sensors, such as dLight1 or iGluSnFR might be needed in future studies for a better interpretation of the peak parameters and oscillation phenotypes in spheroids, as well as the contribution of each cell type in local field activity^36–38^. Nonetheless, the results from this study support the use of calcium activity as a primary readout for HTS using neural spheroids given it’s robust and reproducible phenotypes.

We focused on two specific brain regions and created spheroids with neuronal subtype distributions modeling the human prefrontal cortex (PFC-like spheroids) and ventral tegmental area (VTA-like spheroids), given the role that these two brain regions play in neurological diseases including OUD and Alzheimer’s disease (AD). The use of patient-derived cells or genetically modified cells relevant to specific neurological diseases will be critical for the development of personalized cellular models for precision medicine. As such, we first developed a disease model for AD by incorporating genetically engineered GABAergic neurons carrying the apolipoprotein e4/4 (APOE4/4) allele, which is the strongest genetic risk factor for developing AD in humans, into PFC-like spheroids given that AD leads to neurodegenerative of neocortical regions^39^. Symptoms of AD such as altered cognitive functioning and information processing are associated with altered GABAergic signaling^40^, and in this study, we found baseline disruptions in peak count, width, and synchronicity in PFC-like spheroids with APOE4/4 GABAergic neurons. Although there is a gap of knowledge on how the APOE4/4 allele specifically affects cortical calcium activity and synchronicity in vivo, several lines of evidence support the contribution of GABAergic dysfunction in reducing synchronous neural activity, potentially by altering the network excitation/inhibition balance^40–44^. Given that GABAergic dysfunction is commonly found in the frontal cortex of AD patients carrying the apolipoprotein epsilon 4 allele and in animal models of AD with APOE4 knock-in, it is possible that the abnormal calcium oscillation in PFC-like spheroids with APOE4/4 GABAergic neurons is a result of altered GABAergic transmission^40–42,45–47^.

To be able to use 3D tissues for disease modeling and drug discovery, it is critical to establish their context of use, that is, what stages and mechanisms underlying diseases are reported in a particular assay system, and therefore, what targeted pharmacologically agents will perturb the activity of the system^48,49,50^. Here, we used a panel of drugs that are being used to treat symptoms of AD, with different mechanisms of action (MOA), to assess which targeted pharmacological perturbations reversed the disease phenotypes in our disease spheroid models. In this context, it is worth noting that the study is designed to identify compounds capable a phenotypic rescue, rather than specifically identify compounds that are specific to a single gene target, for example. We tested five compounds including Memantine, Donepezil, two clinically approved compounds used to treat AD, and EUK-134, a preclinical compound. Three compounds (Memantine, Donepezil, and EUK-134) were able to reverse the AD disease phenotypes following our acute treatment protocol. While these compounds treat the symptoms of AD rather than the underlying disease, a recent meta-analysis showed that Memantine, which blocks NMDA receptors, has shown credible efficacy when prescribed alone or in combination with cholinesterase inhibitors such as Donepezil^51,52^. In this model, we hypothesize that although Memantine likely does not target GABAergic transmission, it may still rescue the disease calcium phenotype observed in the APOE4/4 GABAergic PFC-like spheroids via alteration of the (APOE3/3) glutamatergic activity. Given their MOAs, the observed reversal of disease phenotype in PFC-like spheroids with APOE4/4 GABAergic neurons is likely a result of excitation/inhibition rebalancing within the spheroid’s neural network. We also found that EUK-134 was able to reverse deficits in our APOE4/4 PFC-like spheroids within the 90-minute treatment window, which may be reflective of its MOA in oxidative stress. Interestingly, EUK-134 has been associated with reduced beta-amyloid plaques in mouse models of AD^53,54^. Although we did not observe increased beta-amyloid plaque formation in the APOE4/4 PFC-like spheroids during the 3-week culture, it is possible that longer culture times may allow for detectable beta-amyloid plaque formation. Future work to investigate how these drugs are causing a phenotypic rescue, and whether that translates to a symptomatic rescue, will be important to consider.

Further studies are underway to assess the presence of clinical hallmarks of AD to further establish its context of use and mechanistically explain the different pharmacological responses obtained by each drug. Importantly, we expect the assay to be sensitive to multiple MOAs to include non-direct protein targeting disease modulators (e.g., the NMDA antagonist memantine, and the cholinesterase inhibitors rivastigmine and donepezil), and the dose-dependent effect of each compound tested should be assays in both WT and diseased spheroids to investigate potential polypharmacology, specificity, off-target effects, and toxicity.

To model OUD, we tested PFC- and VTA-like spheroids given the role these two brain regions play in drug reward, withdrawal, and relapse^55^. PFC- and VTA-like spheroids were chronically treated with DAMGO, a MOR agonist, for 10 days prior to calcium activity recordings, with and without a 3-day washout period mimicking withdrawal. In line with clinical imaging studies showing alterations in magnetic resonance imaging (MRI) signal in the PFC and VTA of people with OUD^56,57^, we found changes in baseline activity in both spheroid types, where chronic DAMGO treatment in PFC-like spheroids reduced peak count while in VTA-like spheroids chronic DAMGO and DAMGO withdrawal increased peak count. Interestingly, treatment with naloxone, an MOR antagonist used to reverse opioid overdoses in humans, reversed DAMGO-induced reductions in peak count in PFC-like spheroids while having no impact on VTA-like spheroids, suggesting that the mechanism may be through glutamatergic and GABAergic transmission more than dopaminergic in these spheroids.

In conclusion, we have developed a highly reproducible functional neural spheroid assay platform where cell-type composition can be adjusted to mimic a specific brain region of interest and that can be used for HTS and neural circuitry modeling. We also developed disease models for AD and OUD, and machine learning classifier models were used to quantify the predictability of disease phenotypes, showing that the AD model showed a highly predictive disease-related phenotype. Although the drug treatments shown here were limited to 90 min, future studies will also investigate the effect of long-term and repeat dosing protocols on neuronal spheroids which may be necessary for certain drugs’ MOA. The successful incorporation of genetically encoded biosensors such as GCaMPf6 in this neural spheroid models further allows continuous monitoring of neuronal activity. In addition, we show that our methodology can be used to fuse neural spheroids to create assembloids and establish neural circuitry. It is worth noting that there remain intrinsic limitations of this system, however. In particular, while the use of differentiated neural cells presents a unique advantage in this system as described above, the lack of neural progenitor cells and in vivo-like spatial organization will limit their use for modeling neurological development, for which the use of brain organoids remains critical. Future studies are on-going aimed to further establish the context of use of these neural disease models for drug discovery and development by doing a more in-depth phenotypic and genomic analysis of disease biomarkers, pharmacological profiling with additional compounds of relevant targets and mechanisms, and development of high-throughput methods for making neural circuit-specific assembloids to study circuit-level effects of compounds on neurological diseases. Overall, the current study lays the groundwork for potentially improving drug discovery for neurological diseases by creating physiological and pathological relevant tissue models that are robust and have the throughput for drug discovery and development.

## Methods

### Cells and donor information

Differentiated and matured human-induced pluripotent stem cells (iPSC) derived from healthy and diseased neurons and astrocytes were obtained from FujiFilm CDI. Wild-type (Wt) cell lines used in this study including dopaminergic neurons (iCell DopaNeurons, or “Dopa”, cat# R1088, donor ID 01279, lots 105288, 102614, and 105307), glutamatergic neurons (iCell GlutaNeurons, or “Gluta”, cat# R1061, donor ID 01279, lots 105449 and 103289), GABAergic neurons (iCell GABANeurons, or “GABA”, cat# R1013, donor ID 01434, lots 105447), and astrocytes (iCell Astrocytes, or “Astro”, cat# R1092, donor ID 01434, lot 105337 and 105993). Additionally, APOE4 GABAergic neurons (iCell GABANeurons AD APOE E4/E4, cat# R1168, donor ID 01434, lots 100623 and 104950) which encode the apolipoprotein E gene (APOE) were used for the Alzheimer’s disease spheroids (AD).

Cell lines with donor ID 01279 come from a healthy male age 50–59, while cell lines with the donor 01434 come from a healthy female <18 years old. Lot COAs are available on https://www.fujifilmcdi.com/coa-lookup. We also stained thawed cells after 3 weeks of culture for neuronal subtype markers as an internal quality control of the cell source quality, and checked for activity by MEA, which is shown in Supplementary Figs. S3 and S4.

### Tissue culture media

Cells thawed and resuspended in base media that differed by cell type were used to create a cell suspension: for dopaminergic neurons, iCell Base Medium 1 (FujiFilm CDI, cat# M1010) supplemented with 2% Neural Supplement B (FujiFilm CDI, cat# M1029) plus 1% Nervous System Supplement (FujiFilm CDI, cat# M1031) was used as base media. For GABAergic neurons or astrocytes, iCell Base Medium 1 supplemented with 2% Neural Supplement A (FujiFilm CDI, cat# M1032) was used. For glutamatergic neurons, BrainPhys Neuronal Medium (Stem Cell Technologies, cat# 05790) supplemented with 2% Neural Supplement B, 1% Nervous System Supplement, 1% N2 supplement (Thermo, cat# 17502048), and 0.1% laminin (Invitrogen, cat# 23017-015) was used.

Maintenance media was the same for all spheroids and consisted of BrainPhys Neuronal Medium (Stem Cell Technologies, cat #05790) supplemented with 1× N2 supplement, 1× B27 (Thermo, cat# 17504), 20 ng/mL brain-derived neurotrophic factor and 20 ng/mL glial cell line-derived neurotrophic factor (BDNF and GDNF; Stem Cell Technologies, cat# 78005 and 78058, respectively), 1 µg/mL laminin (Invitrogen, cat# 23017-015), 1 mM cyclic adenosine monophosphate (cAMP; Tocris, cat# 1141), and 200 nM ascorbic acid (Tocris, cat# 4055). A stock solution consisting of all materials except cAMP and ascorbic acid was prepared in advance, while cAMP and ascorbic acid were added to the media fresh on each day of media changes.

### Cell thawing

For thawing, GABAergic neurons and astrocytes were placed in a 37 °C water bath for 3 min while dopaminergic and glutamatergic neurons for 2 min, according to the manufacturer’s instructions. The contents of each vial were dispensed into separate 15-mL conical tubes. Base media (1 mL) for each cell type was added to the empty cell vials to collect any remaining cells, which is then dispensed in drop-wise fashion on top of the cell suspension in each tube. An additional 8 mL of base media was added to each 15-mL conical tube before gently mixing the cell suspension. Tubes containing cell suspension of either GABAergic neurons or astrocytes were centrifuged at 300 × *g* × 5 min, while tubes with either dopaminergic or glutamatergic neurons were centrifuged at 400 × *g* × 5 min. The supernatant was aspirated and resuspended in 2 mL of base media, then cells for each cell type were counted using a Countess Cell Counter (Thermo). For 2D neuron cultures, neurons were thawed and seeded in a 96-well plate (80,000 cells/well for glutamatergic neurons, 64,000 cells/well for dopaminergic neurons, and 40,000 cells/well for GABAergic neurons) according to the manufacturer’s instructions. Neurons were fixed after 3 weeks of culturing and immunostained for neuronal markers.

### Generation of spheroids

After counting, base media was added to achieve a cell suspension containing 5e5 cells/mL for each cell type. Cell types required in each spheroid type were then mixed in fresh 50 mL conical tubes, and base media of the majority cell type was added to achieve a final concentration of 2e5 cells/mL. The cell-type compositions of control spheroids include 100% Dopa, 100% Gluta, 100% GABA, 90% Dopa + 10% Astro, 90% Gluta + 10% Astro, 90% GABA + 10% Astro. A proof-of-concept study (Fig. 3) generated 16 types of spheroids consisting of 90% neuron and 10% astrocyte but with differing percentages of neuronal subtypes, and these spheroid compositions can be found in the figures. Brain region-specific spheroids modeling the ventral tegmental area (VTA-like) and prefrontal cortex (PFC-like) each contained 90% neurons + 10% astrocytes with differing neuronal cell-type compositions. VTA-like spheroids contained 65% Dopa, 5% Gluta, and 30% GABA neurons while PFC-like spheroids contained 70% Gluta + 30% GABA neurons. In total, 50 µL mixed cell suspension containing 1e4 total viable cells was manually dispensed with a 16-channel multichannel Finnpipette (Thermo) into 384-well round bottom ultra-low attachment (ULA) spheroid microplates (Corning, cat# 3830). Plates were then sealed with parafilm and centrifuged at 300 x g for 10 min to pull cells to the bottom of the plate. One day later, 5 µL of base media was removed and 45 µL maintenance media was added to achieve 90 µL total media each well. Half-media change using the maintenance media was done every other day prior to testing.

### Generation of assembloids

Assembloids were made 1-week prior to recording by placing one of each spheroid type into one 1.7-mL tube together. Both spheroids were pulled up into a wide bore 200-µL pipette tip (Rainin, cat# 30389188) with only 15 µL media and dispensed into the bottom of a well of the Corning ULA round bottom plates (#3830). Collagen I (Fisher, cat# CB354249) was made with media, 10× phosphate-buffered saline, and 1 N NaOH at a 3 mg/mL concentration and 30 µL was pipetted on top of the two spheroids. The plate was centrifuged at 300 × *g* × 2 min and placed in the incubator at 37 °C for overnight gelling and the following day, 50 µL media was added to each well. Maintenance media used was the same as spheroids and half-media changes were performed every other day prior to testing.

### Viruses and dyes

To assess calcium activity, the calcium dye, Cal6 (Molecular Devices, cat# R8190), or virus-mediated expression of the genetically encoded calcium indicator, GCaMP6f was used. Each vial of Cal6 was dissolved according to the manufacturer’s instructions in 10 mL maintenance media. Two hours before the activity was recorded, half of the spheroid media was exchanged for media with Cal6. The plates were covered in foil and placed in the incubator at 37 °C during a 2-h incubation period before calcium recording using a fluorescent imaging plate reader (FLIPR) or a confocal (Opera Phenix Plus High-Content Imaging System). For virus-mediated GCaMP6f expression, the adeno-associated virus (AAV) serotype 9 with CAG promoter (AAV9-CAG-GCaMP6f-WPRE-SV40, Addgene, cat# 100836-AAV9) was used to express GcaMP6f in both neurons and astrocytes. AAV was added to the media on day 7 to allow for 2 weeks of expression prior to recording or testing at 2e5 multiplicity of infection (MOI). Spheroids transduced with GCaMP6f were protected from light and their activities were recorded using the confocal (Opera Phenix Plus High-Content Imaging System).

Designer receptors exclusively activated by designer drugs (DREADDs) viruses were used to stimulate or inhibit neuronal activity within spheroids^58^. Both viruses were retrograde AAVs expressed under the human Synapsin promoter for expression in neurons and fused with an mCherry fluorophore. The DREADDs viruses either inserted the designer receptor hM4D(Gi), an inhibitory G-protein coupled receptor, or hM3D(Gq), a stimulatory G-protein coupled receptor (custom-made Chemogenetics AAV: Addgene). pAAV-hSyn-hM4D(Gi)-mCherry and pAAV-hSyn-hM3D(Gq)-mCherry was a gift from Bryan Roth (Addgene viral prep # 50475-AAV9 and # 50474-AAV9). Clozapine-N-oxide (CNO, Tocris cat# 4936) was suspended in dimethyl sulfoxide (DMSO) and used as the designer drug to activate the DREADDs viruses. CNO was tested at 1 and 10 μM, with data reported from the 1 μM concentration.

### Fluorescent imaging plate reader (FLIPR) measurements

The FLIPR Penta (Molecular Devices) was used to assess calcium fluorescence across the 384-well plate simultaneously and to observe changes after treatment with compounds. The evening before calcium activity was measured, plates were centrifuged at 300×*g* for 2 min to get spheroids to the bottom of the well in a centered position. On the day of recording, the plate used for recording was placed in the read plate position inside of the FLIPR following the 2-h Cal6 incubation at 37 °C. Standard filter sets were used for Cal6 imaging with excitation set at 470–495 and emission at 515–575 nm. Fluorescent image reads were taken with frame rates of 1.67 frames per second (fps) for all plates, with exposure time of 30 ms and 50% excitation intensity. Recordings from the initial seven plates consisted of 1000 reads (10-min recordings) with 2.5 gain, while the final two plates consisted of 500 reads (5-min recordings) with a gain of 2. Baseline recordings were taken across all plates and, if applicable, more recordings were obtained 1-, 30-, 60-, and/or 90-min after compound treatment. In between recordings, plates were wrapped in foil and placed back in the incubator at 37 °C, 5% CO_2_.

### Confocal imaging

The Opera Phenix Plus High-Content Imaging System (Perkin Elmer) spinning disk confocal was used to record calcium activity from live spheroids in individual wells. Prior to recording, the stage was pre-warmed to 37 °C, and the carbon dioxide was set to 5%. Recordings were obtained both from spheroids expressing GCaMP6f along with those incubated in Cal6 dye. Recordings were captured with a 20× water immersion objective 55 μm from the bottom of the well, and were obtained at a frame rate of 1.6 fps with 480 frames total, making the recordings 5-min. The protocol was set to record well to well such that recordings were automated but taken from one spheroid at a time before recording from subsequent wells. For all calcium activity recordings obtained from the Phenix Plus, the FITC channel was used where excitation was set to 488 nm and emission at 535 nm. For spheroids expressing GCaMP6f, the exposure time was set to 20 ms and the laser power was set to 30% while for spheroids in Cal6 dye, the exposure time was set to 20 ms and the laser power was set to 10%. The focal plane for recordings from assembloids varied depending on where they were suspended in the collagen, though these recordings were all obtained within 250 µm from the bottom of the well. For assembloids, the exposure time was set to 40 ms with laser power set to 40%. For fixed and optically cleared spheroids, we used the Leica TCS SP8 Spectral Confocal system, 25×/0.95 water objective, with 0.57- or 0.8-micron Z-step for cell-type marker quantifications, or 10 micron Z-steps for disease marker quantifications.

#### MEA preparation and recording

In all, 48-well MEA plates (Axion Biosystems) were prepared according to the manufacturer’s protocol. Briefly, plates were precoated with either 0.1% or 0.07% polyethyleneimine for 1 h at 37 °C depending on neuronal cell type, then rinsed three times with DPBS and three times with sterile water and allowed to air dry in a biosafety cabinet overnight. The following day, neurons were thawed and centrifuged according to the manufacturer’s instructions. Cell pellets were resuspended in a small volume of the complete maintenance medium recommended for that neuronal cell type and supplemented with 10 µg/mL laminin. However, iCell GlutaNeurons were resuspended using medium supplemented with 100 µg/ml laminin as per the manufacturer’s protocol. In addition, before plating iCell DopaNeurons, an additional precoating step was performed with 80 µg/ml laminin at 37 °C for 30 min after the initial PEI coating. Finally, cell suspension droplets were dispensed directly over the recording electrode area of the well of the 48-well MEA at the manufacturer’s recommended seeding density for each cell type. Following a 60-min incubation at 37 °C to allow cells to settle in the MEA plates, the appropriate maintenance medium for each cell type was added to each well at a final volume of 300 µl per well. Neurons were maintained for 3 weeks with 50% media changes occurring every 2–3 days. Neuronal activity was recorded after 3 weeks using the MaestroPro system (Axion Biosystems) and AxIS software v2.1–2.5 (Axion Biosystems). To analyze the data, the AxIS Navigator software was used to convert the AxIS Raw recording output file to an AxIS Spike file which was then loaded into the Neural Metric Tool. In the Neural Metric Tool, the “Envelope” burst detection method was selected with a “Threshold Factor” set to 3, the “Min Electrode” parameter set to 50%, and the “Burst Inclusion” parameter set to 75%. The software then generated raster plots, analyzed network bursts, and calculated synchrony metrics which were exported as .csv files and used to generate the necessary plots.

### Chronic DAMGO treatment

To model OUD, a subset of spheroids was treated with [D-Ala2, NMe-Phe4, Gly-ol5]-enkephalin (DAMGO; Tocris, cat# 1171), a selective µ-opioid receptor (MOR) agonist, chronically during the 3-week spheroid maintenance period. DAMGO was reconstituted in water at a 1 mM concentration and diluted in media to 20 μM for a final concentration of 10 μM after the half-media exchange. For spheroids subjected to chronic DAMGO treatment, 20 μM DAMGO in maintenance media was added via half-media exchange beginning on day 10, with treatments occurring every other day for 10 days, giving a total of five treatments. For spheroids subjected to model DAMGO withdrawal, the same protocol was followed except that spheroid did not receive DAMGO for the final treatment and instead were subjected to a three-day washout period.

### 384 Pin tool compound addition

A 384-well pin tool (Rexroth) was used to transfer compounds simultaneously to the spheroid plate. Compound transfer via the 384-well pin tool occurred after the baseline recordings with the FLIPR. In total, 60 nL of compound suspended in DMSO was transferred to 60 µL of media in each well. Immediately after compound transfer, the spheroid plate was either placed back inside of the FLIPR for a recording 1-min after compound treatment or placed back in the incubator if post-treatment recording was >30 min after compound transfer.

### 3D cell titer Glo

CellTiter-Glo 3D Cell Viability Assay (Promega, cat# G9681) was used according to the manufacturer’s instructions. CellTiter-Glo 3D Reagent was thawed overnight at 4 °C and brought to room temperature (RT) for 20-min before use. After the FLIPR assay, 30 µL of CellTiter-Glo 3D Reagent was added to the spheroid plate and was mixed by shaking for 5-min at RT followed by a 25-min incubation period on the shaker at RT. Luminescence was read using a PHERAstar FSX microplate reader (BMG LabTech) to measure the amount of adenosine 5′-triphosphate (ATP) present.

### Calcein AM and propidium iodide (PI) staining

Imaging of live and dead cells was done via Calcein AM (Thermo, cat# C1430) and PI (Thermo, cat# P3566) staining on live spheroids. Calcein AM and PI were diluted in 1× Dulbecco’s phosphate-buffered saline (DPBS; Thermo, cat# 14040141) to concentrations of 1:2000 and 1:1000 to achieve final concentrations of 0.5 and 1.5 μM, respectively. Half of the media was removed from each well (45 μL) and was exchanged with Calcein AM and PI in DPBS. Spheroids were incubated at 37 °C for 30-min prior to live cell imaging. For imaging, spheroids were placed in the Phenix Plus with the stage pre-warmed to 37 °C and 5% carbon dioxide circulating. The FITC channel, with 488 nm excitation and 535 nm emission, was used to image Calcein AM while Cy3 (excitation of 530 nm, emission of 620 nm) was used to image PI. 150 image stacks were collected with a 10× air objective using a 2 μM z-step.

### Spheroid fixation

Spheroids were fixed with 4% paraformaldehyde (PFA) in PBS overnight at 4 °C. The following day, spheroids were washed with PBS, where half of the PFA was removed and exchanged with PBS, a total of four times. On the final wash, PBS with 0.1% sodium azide (Sigma, cat# S2002) was added for spheroid preservation. Plates were sealed with parafilm and stored at 4 °C until further use.

### Immunohistochemistry (IHC) of whole spheroids

IHC was used to co-stain for (1) neurons and astrocytes, (2) neuronal cell-type markers, (3) pre- and postsynaptic markers, and (4) disease markers. For (1), polyclonal chicken anti-MAP2 (Abcam, cat# ab5392, 1:500) was used to label neurons while astrocytes were stained with rabbit polyclonal anti-GFAP antibody (Abcam, cat# ab7260, 1:500). For (2) neuronal cell-type markers, polyclonal chicken anti-Tyrosine Hydroxylase (Abcam, cat# ab76442, 1:500) was used to stain dopaminergic neurons, monoclonal mouse anti-vGluT1 (Abcam, cat# ab242204, 1:250) was used to stain glutamatergic neurons, and polyclonal rabbit anti-parvalbumin (Abcam, cat# ab11427, 1:500) was used to stain GABAergic neurons. For (3), mouse monoclonal anti-bassoon antibody (Abcam, cat# ab82958, 1:100) was used as a presynaptic marker while rabbit polyclonal anti-homer1 antibody (Abcam, cat# ab97593, 1:100) was used as a postsynaptic marker. Secondary antibodies used in this study include goat anti-mouse Alexa Fluor 488, goat anti-rabbit Alexa Fluor 488, goat anti-chicken Alexa Fluor 568, goat anti-rabbit Alexa Fluor 568, goat anti-mouse, Alexa Fluor 568, goat anti-rabbit Alexa Fluor 647, goat anti-chicken Alexa Fluor 647, and goat anti-mouse Alexa Fluor 647 (Invitrogen, cat# A28175, A11008, A11041, A11011, A11004, A32733, A32933, A32728, respectively).

For the immunostaining assay, all liquid removal steps were performed via manual pipetting and all incubation steps occurred on a shaker. PBS with 0.1% sodium azide was removed and blocking solution consisting of 5% normal goat serum (NGS; Millipore Sigma, cat#S26-LITER), 2% bovine serum albumin (BSA; Fisher, cat# BP1605), and 0.5% Triton X-100 (Sigma, cat# X-100) in PBS was added to spheroid containing wells for a 30-min incubation at room temperature. After 30 min, half of the blocking solution was removed, and primary antibodies made in equal volume of blocking solution with double the desired concentration were added for final concentrations as indicated above. Spheroids were incubated with primary antibodies overnight at 37 °C on a shaker, with the exception of anti-Homer and anti-bassoon which required 72 h of incubation at 37 °C. Spheroids were then washed with PBS + 0.3% Triton X-100 in three half changes followed by an additional three washes with 15-min incubations. Secondary antibodies were made in blocking solution and were added via a half buffer change to achieve a final staining concentration of 1:600. Spheroids were incubated with secondary antibodies at 37 °C overnight. Hoechst 33342 (Thermo, cat# 62249, 1:10,000) was used for nuclei staining and washes occurred the same as described above with primary antibodies.

### Immunofluorescence staining for spheroid cryosections and 2D cultures

For immunofluorescence staining, samples were first incubated in blocking solution (5% NGS, 1% BSA, and 0.7%Triton in PBS) for 1 h in room temperature, then incubated overnight at 4 °C with primary antibodies diluted in blocking solution. The next day, the primary antibody was aspirated and the samples were washed three times with PBST (0.7% Triton) before a 2 h incubation at room temperature in secondary antibodies diluted in blocking solution. After 3 washes using PBST, samples were counterstained with Hoechst to visualize the nuclei. Slides with cryosections were mounted using ProLong Gold antifade reagent (Invitrogen) while 2D cultures were stored in PBS until imaging.

The following primary antibodies were used for immunostaining: vGluT1 (ab242204, 1:200), NSE (ab834, 1:25), TH (ab76442, 1:500), MAP2 (ab5392, 1:1000), GABA (A2052, 1:500), GFAP (ab7260, 1:500), and NeuN (MAB377, 1:25). The following secondary antibodies were used at 1:1000 dilution: goat anti-mouse Alexa Fluor 488, goat anti-rabbit Alexa Fluor 569, goat anti-chicken 647.

### Cryosection protocol

Fixed spheroids were cryopreserved in 15% sucrose solution overnight, then transferred to a 30% sucrose solution, and kept at 4 °C. After the spheroids sink, they were embedded in Tissue-Tek OCT medium. Subsequently, 30-μm-thick sections were obtained using a cryostat and mounted directly on super-frosted slides. Slides were air-dried and then washed with PBS to remove excess OCT.

### Fluorescent in situ hybridization (FISH)

FISH was performed to validate glutamatergic cell-type compositions in brain region-specific designer spheroids modeling the VTA and PFC. Homo sapiens solute carrier family 17 (vesicular glutamate transporter) member 7 mRNA (Hs-SLC17A7, cat# 415611; GenBank Accession Number: NM_020309.3) was used to probe for glutamatergic neurons according to the RNAScope Multiplex Fluorescent Reagent Kit v2 user manual (Advanced Cell Diagnostics, cat# 323100). The tissue was pre-treated according to the formalin-fixed paraffin-embedded (FFPE) preparation method, without the deparaffinizing step. Following hydrogen peroxide incubation, spheroids were washed with DI water and incubated in RNAscope Protease Plus Reagent (supplied in above kit) for 30-min at 40 °C. Protease plus reagent was removed with DI water and the vGluT1 (SLC17A7) was assigned to channel 2 (C2), noted as SLC17A7-C2. After hybridization of the probes, pre-amplification and amplification reagents supplied in the kit were applied according to the user guide, where amplification 1 reagent (AMP1) was added for 30-min at 40 °C followed by amplification reagent 2 (AMP2) was added for 30-min at 40 °C and amplification reagent 3 (AMP3) was added for 15-min at 40 C. The fluorescent Opal 570 dye was added to channel 2 containing SLC17A7-C2 (excitation: 550 nm, emission: 570 nm; Akoya Biosciences). DAPI (Invitrogen, cat# D1306) was added in the final step to spheroids for 30 s, then washed with PBS. Spheroids remained in PBS until tissue clearing reagent was added. Tissue was washed in 1× wash buffer twice for 2-min each time between incubations after probe hybridization steps. Prior to probe hybridization, spheroids were washed with DI water in accordance with the manufacturer’s instructions.

### Tissue clearing of whole spheroids

After immunostaining or FISH, ScaleS4 Tissue clearing solution was added to spheroids to reduce autofluorescence during image acquisition, as previously described^59^. ScaleS4 was made with 40% d-sorbitol (Sigma, cat# S6021), 10% glycerol (Sigma, cat# G2289), 4 M Urea (Sigma, cat# U5378), 0.2% triton X-100, 15% DMSO (Sigma, cat# D2650) in UltraPure water (Invitrogen, cat# 10977-015). ScaleS4 solution was mixed via shaking at 37 °C for 2 days and stored at 4 °C until future use. Before the clearing solution was added, all PBT for IHC spheroids or all wash buffer for FISH spheroids was removed from the well. When nuclear staining is needed, Hoechst 33342 (Thermo, cat# 62249) was added at a 1:2000 dilution in the ScaleS4 clearing solution, and 60 µL of clearing solution with or without Hoechst was added to each well of the 384-well plate containing spheroids. Spheroid plates were wrapped in foil and placed on the shaker at 37 °C overnight. The following day, the plate was sealed with parafilm and placed at 4 °C until imaging.

### Graphical plots

For time series plots, heatmap plots, correlation matrices, and radar plots, Python 3.8 was used. Specifically, the packages matplotlib and seaborn were used to generate radar plots, heatmaps, time series plots, and correlation matrices^60,61^. The Python packages, pandas and numpy, were used to normalize data prior to graphically plotting^62,63^. For principal component analysis scatter plots, TIBCO Spotfire was used, and for column graphs, GraphPad Prism 9.1 Software was used. Schematics used in Figs. 1, 3, 4, and 7 were created with Biorender.

### Calcium activity analysis

Calcium oscillatory peak detection data from FLIPR recordings was obtained through ScreenWorks 5.1 (Molecular Devices). Initial peak detection analysis occurred within ScreenWorks 5.1 via the PeakPro 2.0 module. Here, all parameters were set to be the same for all wells per plate. The event polarity was always set to positive and the search vector length was always set to 11. The baseline, trigger level, which is automatically set to 10% above the baseline, and dynamic threshold, which is the threshold for peak detection were automatically identified by the PeakPro 2.0 module. Wells were manually checked to ensure these parameters were accurately identified prior to analysis. After analysis, data from 17 peak parameters (mean peak amplitude, peak amplitude standard deviation (SD), peak count, mean peak rate, peak rate SD, peak spacing, peaking spacing SD, mean number of early-depolarization like event (EAD-like) peaks per well, calcium transient duration (CTD) at 50% (peak width at 50% amplitude), CTD at 90% (peak width at 90% amplitude), rise slope, rise slope SD, mean peak rise time, peak rise time SD, decay slope, decay slope SD, mean peak decay time, and peak decay time SD) was exported to a STATALL file that could be converted to a Microsoft Excel spreadsheet. The percent coefficient of variance (%CV) was calculated within each plate similar to the previously described^20^ to measure variability of each exported parameter. Parameters were included in future analysis if they were under the threshold cutoff of 30% CV, which included peak count, peak rate, peak spacing, peak width at 50% and 90% amplitude, peak amplitude, peak rise time, peak decay time, rise slope, and decay slope (Supplementary Table S1). All data was normalized to the average of DMSO-treated control wells within each plate. Each group represented on radar plots shows the mean in comparison to DMSO vehicle controls, which should always average to 100%. Bar plots with individual values are reported as mean ± SEM.

For peak detection data obtained from the Phenix Plus confocal microscope, image sequences were stored in and exported from Columbus Image Data Storage and Analysis as single-plane TIFFs. Each recording was imported into ImageJ and converted to a single stack. Prior to peak detection analysis, the T-function, F div F0, in ImageJ was used to obtain calcium signals normalized to background fluorescence. The ImageJ Plugin, LC_Pro, was used to automatically identify regions of interest containing dynamic calcium signals across the image sequence^64^. The automated analysis was used on the F div F0 recording so that calcium measurements would be reported as normalized fluorescent values (F/F0). For the LC_Pro analysis, default settings were used. F/F0 values for a region of interest (ROI) were exported if they contained a high signal-to-noise ratio and exported to a text file titled ROI Normalized. Once this text file was converted to a csv file, it was uploaded into Python for peak detection analysis. While code for this analysis is publicly available on GitHub, a brief description of the process is described. First, data from all identified ROIs was normalized such that the minimum F/F0 value was equal to 1. Time series plots showing mean signal plus variability represented as 95% confidence interval were generated, along with heatmaps showing activity across every identified ROI in the time series, and a correlation matrix plotted as a heatmap representing correlation coefficients across all ROIs. The correlation matrix was used to describe how synchronous the calcium activity within a spheroid was, and a synchrony score was measured by calculating the average correlation coefficient across the matrix. Given that LC_Pro identifies different numbers of ROIs for each recording, we used a random sample generator to randomly choose 12 ROIs for peak detection analysis. To ensure this random sample reflected the population activity, we required the correlation score of the random sample to be within 5% of the correlation score of the population of ROIs. The find_peaks package was imported from scipy.signal and used for peak detection analysis. The scipy package, find_peaks, was used to detect and measure peak parameters including peak count, amplitude, and width^65^.

### Image analysis

All image intensity quantitation was carried out in Fiji. To compare average intensity between spheroids for selective markers, the volume of the spheroid was calculated by first summing all the fluorescence channels together, and then manually choosing an intensity threshold for the sum image that selectively rejected the background. Applying this threshold generated a binary mask for the spheroid that was further manually edited to reject debris, scatter/weak fluorescence from the well walls, etc. The final, edited binary mask was then applied to all fluorescence channels to zero out any signal not originating from the spheroid. The total number of pixels in the edited binary mask was taken as the volume of the spheroid (in pixels). The volume-averaged fluorescence intensity in each fluorescence channel was calculated as the integrated intensity from the entire masked image volume, divided by the volume of the spheroid.

Nuclear segmentation for nuclei quantification in 3D models: segmenting cells in 3D microscopy images provides a much greater challenge compared to 2D segmentation as (1) the exact boundaries of cells in 3D images are not always well defined; (2) low signal-to-noise ratios and dense packing of nuclei are in typical microscopy images. In this work, we use a recent prominent deep learning algorithm, StarDist for nuclei segmentation^66,67^. StarDist 2D trains a convolutional neural network (CNN) to localize cell nuclei^66^. For every pixel inside a nucleus, StarDist predicts a star-convex polygon parameterized by the radial distance to the nucleus boundary. In addition, it also predicts an object probability to determine which pixels are part of cell nuclei. StarDist 3D further extends the 2D approach to 3D volumes and uses star-convex polyhedral for cell nuclei^67^. For 2D nuclear segmentation, we used the StarDist plugin for Fiji with a “Versatile” pre-trained model, and the default NMS Postprocessing parameters of Probability/Score Threshold = 0.5, Overlap Threshold = 0.45. For 3D nuclear segmentation, we used the StarDist napari plugin (https://github.com/stardist/stardist-napari) for 3D nuclear segmentation. Pre-trained model (3D_demo) and default settings (U-Net architecture, prob_thresh=0.707933, nms_thresh=0.3) are used for all datasets.

### Statistics and reproducibility

Python 3.8, R Studio 4.0.3, and GraphPad Prism 9.1 were used for statistical analysis. Principal component analysis was performed in Python using the package, scikit-learn^68^, and the number of components was determined based on retaining >85% of variance. Prior to quantifying predictive accuracy of activity phenotypes in neurological disease models with a random forest classifier (RFC) machine learning model, training and test datasets were generated which contained 80% and 20% of the data, respectively. A *t* test was used to ensure no significant differences between data in the training versus the test sets, and *z* tests were used to ensure similar frequencies of each genotype or pre-treatment group were represented. After confirming no significant differences between training and test datasets, the PCA data in the test dataset was tested against the RFC model and labeling accuracy for each genotype or pre-treatment group are reported. Comparisons between two groups were analyzed with unpaired *t* tests when comparing separate groups of spheroids, and paired *t* tests when examining effects within the same spheroid. Comparisons between two groups or more were analyzed with one-way ANOVA, and significant main effects were followed up with Dunnett’s multiple comparisons tests. Data are reported as mean ± SEM for column and violin plots, and as mean for radar plots. Significance was set at *P* < 0.05. Outliers were identified using Grubbs’ test at alpha=0.05. Data used in the current study spanned ten 384-well plates. Data from Fig. 3 consisted of 16 technical replicates per group from one independent experiment. Data from Figs. 4–6 were run over three independent experiments, with technical replicates consisting of *n* > 3 per experiment. Data from Fig. 7b, c was collected from three independent experiments with *n* = 8–12 technical replicates per experiment. Data from Fig. 7d–f was collected from two independent experiments with *n* = 4–9 technical replicates per experiment. Exact details are listed in figure legends

### Reporting summary

Further information on research design is available in the Nature Portfolio Reporting Summary linked to this article.

### Supplementary information

Supplementary Information

Description of Supplementary Materials

Supplementary Data

Video S1_VTA-like stain

Video S2_PFC-stain

Video S3_VTA-like spheroid

Video S4_PFC-like spheroid

Video S5_wtGABA_SNS_gCAMP6

Video S6_wtGABA_SNS_cal6

Video S7_APOE4-GABA_SNS_gCAMP6

Video S8_APOE4-GABA_SNS_cal6

Reporting Summary

## Data Availability

The data supporting the findings of this study are available within the paper, its Supplementary Information, and are available from the corresponding authors upon reasonable request. The source data behind the graphs can be found in Supplementary Data.

## Code availability

Codes are available on GitHub as described in “Methods” above: https://github.com/stardist/stardist-napari; https://github.com/carolinestrong/LC_Pro.
