# Supplementary material for: Functional brain region-specific neural spheroids for modeling neurological diseases and therapeutics screening
Source: Commun Biol. 2023 Nov 28;6:1211. doi: 10.1038/s42003-023-05582-8 (PMC10684574; doi:10.1038/s42003-023-05582-8)
Supplement: Supplementary file 2 — Description of Supplementary Materials [file 42003_2023_5582_MOESM2_ESM.docx]

**Description of Additional Supplementary Files**

**File name:** Video S1_VTA-like stain

**Description:** Video showing confocal imaging through a VTA-like spheroid stained for nuclei and neural markers TH, vGluT1, and parvalbumin.

**File name:** Video S2_PFC-stain

**Description:** Video showing confocal imaging through a PFC-like spheroid stained for nuclei and neural markers TH, vGluT1, and parvalbumin.

**File name:** Video S3_VTA-like spheroid

**Description:** Video showing live confocal imaging of calcium activity (Cal6) in a VTA-like spheroid. Live images were taken using a frame rate of 1.6 frames per second. Video was generated using a frame rate of 7 frames per second.

**File name:** Video S4_PFC-like spheroid

**Description:** Video showing live confocal imaging of calcium activity (Cal6) in a PFC-like spheroid. Live images were taken using a frame rate of 1.6 frames per second. Video was generated using a frame rate of 7 frames per second.

**File name:** Video S5_wtGABA_SNS_gCAMP6

**Description:** Video showing live confocal imaging of calcium activity (gCAMP6) in two spheroids comprised of healthy GABAergic neurons and astrocytes. Live images were taken using a frame rate of 1.6 frames per second. Video was generated using a frame rate at 21 frames per second.

**File name:** Video S6_wtGABA_SNS_cal6 Description: Video showing live confocal imaging of calcium activity (Cal6) in two spheroids comprised of healthy GABAergic neurons and astrocytes. Live images were taken using a frame rate of 1.6 frames per second. Video was generated using a frame rate at 21 frames per second.

**File name:** Video S7_APOE4- GABA_SNS_gCAMP6

**Description:** Video showing live confocal imaging of calcium activity (gCAMP6) in two spheroids comprised of APOE4 GABAergic neurons, and astrocytes. Live images were taken using a frame rate of 1.6 frames per second. Video was generated using a frame rate at 21 frames per second.

**File name:** Video S8_APOE4-GABA_SNS_cal6

**Description:** Video showing live confocal imaging of calcium activity (Cal6) in two spheroids comprised of APOE4 GABAergic neurons, and astrocytes. Live images were taken using a frame rate of 1.6 frames per second. Video was generated using a frame rate at 21 frames per second.

**File name:** SupplementaryDataFile1

**Description:** Raw data including outliers for graphs in Strong & Zhang et al.
