# Supplementary material for: Functional brain region-specific neural spheroids for modeling neurological diseases and therapeutics screening
Source: Commun Biol. 2023 Nov 28;6:1211. doi: 10.1038/s42003-023-05582-8 (PMC10684574; doi:10.1038/s42003-023-05582-8)
Supplement: Supplementary file 12 — Reporting Summary [file 42003_2023_5582_MOESM12_ESM.pdf]

Corresponding author(s): Emily Lee & Marc Ferrer

Last updated by author(s): Nov 2, 2023

Reporting Summary

Nature Portfolio wishes to improve the reproducibility of the work that we publish. This form provides structure for consistency and transparency in reporting. For further information on Nature Portfolio policies, see our Editorial Policies and the Editorial Policy Checklist.

Statistics

For all statistical analyses, confirm that the following items are present in the figure legend, table legend, main text, or Methods section.

n/a	Confirmed	
☐	☒	The exact sample size (n) for each experimental group/condition, given as a discrete number and unit of measurement
☐	☒	A statement on whether measurements were taken from distinct samples or whether the same sample was measured repeatedly
☐	☒	The statistical test(s) used AND whether they are one- or two-sided<br>*Only common tests should be described solely by name; describe more complex techniques in the Methods section.*
☐	☒	A description of all covariates tested
☐	☒	A description of any assumptions or corrections, such as tests of normality and adjustment for multiple comparisons
☐	☒	A full description of the statistical parameters including central tendency (e.g. means) or other basic estimates (e.g. regression coefficient) AND variation (e.g. standard deviation) or associated estimates of uncertainty (e.g. confidence intervals)
☐	☒	For null hypothesis testing, the test statistic (e.g. F, t, r) with confidence intervals, effect sizes, degrees of freedom and P value noted<br>*Give P values as exact values whenever suitable.*
☒	☐	For Bayesian analysis, information on the choice of priors and Markov chain Monte Carlo settings
☒	☐	For hierarchical and complex designs, identification of the appropriate level for tests and full reporting of outcomes
☐	☒	Estimates of effect sizes (e.g. Cohen's d, Pearson's r), indicating how they were calculated

Our web collection on statistics for biologists contains articles on many of the points above.

Software and code

Policy information about availability of computer code

Data collection	MaestroPro system (Axion Biosystems) and Screenworks 5.1 (Molecular Devices), Harmony 5.1 (Perkin Elmer)
Data analysis	ScreenWorks 5.1 via the PeakPro 2.0 module, Graphpad Prism 9.1, Python, ImageJ, Spotfire TIBCO, Columbus Image Data Storage and Analysis, FIJI, AxIS software v2.1–2.5

For manuscripts utilizing custom algorithms or software that are central to the research but not yet described in published literature, software must be made available to editors and reviewers. We strongly encourage code deposition in a community repository (e.g. GitHub). See the Nature Portfolio guidelines for submitting code & software for further information.

Data

Policy information about availability of data

All manuscripts must include a data availability statement. This statement should provide the following information, where applicable:

- Accession codes, unique identifiers, or web links for publicly available datasets
- A description of any restrictions on data availability
- For clinical datasets or third party data, please ensure that the statement adheres to our policy

The data supporting the findings of this study are available within the paper, its Supplementary Information, and are available from the corresponding authors upon reasonable request.

Research involving human participants, their data, or biological material

Policy information about studies with human participants or human data. See also policy information about sex, gender (identity/presentation), and sexual orientation and race, ethnicity and racism.

Reporting on sex and gender	n/a
Reporting on race, ethnicity, or other socially relevant groupings	n/a
Population characteristics	n/a
Recruitment	n/a
Ethics oversight	n/a

Note that full information on the approval of the study protocol must also be provided in the manuscript.

Field-specific reporting

Please select the one below that is the best fit for your research. If you are not sure, read the appropriate sections before making your selection.

☒ Life sciences ☐ Behavioural & social sciences ☐ Ecological, evolutionary & environmental sciences

For a reference copy of the document with all sections, see nature.com/documents/nr-reporting-summary-flat.pdf

Life sciences study design

All studies must disclose on these points even when the disclosure is negative.

Sample size	Data used in the current study spanned ten 384-well plates. Data from Figure S2 3 consisted of 16 technical replicates per group from one independent experiment. Data from Figures 43-7 6 was run over three independent experiments, with technical replicates consisting of n > 3 per experiment. Data from Figure 7b-c was collected from three independent experiments with n=8-12 technical replicates per experiment. Data from 6 7d-f was collected from two independent experiments with n =4-9> 2 technical replicates per experiment. Exact details are listed in the figure legends.
Data exclusions	If a plate was contaminated with bacteria it was not included in the paper. If spheroids were not in the detection window at time of recording the wells were counted as empty. Outliers were identified by using Grubbs' test, (the ESD method (extreme studentized deviate)), with alpha value set to 0.05 as is standard.
Replication	Data used in the current study spanned ten 384-well plates. Data from Figure S2 3 consisted of 16 technical replicates per group from one independent experiment. Data from Figures 43-7 6 was run over three independent experiments, with technical replicates consisting of n > 3 per experiment. Data from Figure 7b-c was collected from three independent experiments with n=8-12 technical replicates per experiment. Data from 6 7d-f was collected from two independent experiments with n =4-9> 2 technical replicates per experiment. Exact details are listed in the figure legends.
Randomization	n/a see below
Blinding	for the work done in 384w plates with FLIPR, its not really possible to know which well is what condition for drug treatments during the acquisition because it acquires the entire plate at once until we acquire the data and map it back to the well (it's too many wells). Otherwise we just assign based on how our compound management gives us the compound plates, or its half a plate is disease line, half is not. For assembloids, those are lower throughput so those we were fully aware of which were in each well as we had to add the DREADDS viruses or stimulatory drugs.

Reporting for specific materials, systems and methods

We require information from authors about some types of materials, experimental systems and methods used in many studies. Here, indicate whether each material, system or method listed is relevant to your study. If you are not sure if a list item applies to your research, read the appropriate section before selecting a response.

Materials & experimental systems

n/a	Involved in the study
☐	☒ Antibodies
☐	☒ Eukaryotic cell lines
☒	☐ Palaeontology and archaeology
☒	☐ Animals and other organisms
☒	☐ Clinical data
☒	☐ Dual use research of concern
☒	☐ Plants

Methods

n/a	Involved in the study
☒	☐ ChIP-seq
☒	☐ Flow cytometry
☒	☐ MRI-based neuroimaging

Antibodies

Antibodies used: All antibodies and cat numbers are listed in methods

Validation: *Describe the validation of each primary antibody for the species and application, noting any validation statements on the manufacturer's website, relevant citations, antibody profiles in online databases, or data provided in the manuscript.*

Eukaryotic cell lines

Policy information about cell lines and Sex and Gender in Research

Cell line source(s): *State the source of each cell line used and the sex of all primary cell lines and cells derived from human participants or vertebrate models.*

Authentication: *Describe the authentication procedures for each cell line used OR declare that none of the cell lines used were authenticated.*

Mycoplasma contamination: *Confirm that all cell lines tested negative for mycoplasma contamination OR describe the results of the testing for mycoplasma contamination OR declare that the cell lines were not tested for mycoplasma contamination.*

Commonly misidentified lines (See ICLAC register): *Name any commonly misidentified cell lines used in the study and provide a rationale for their use.*

Plants

Seed stocks: *Report on the source of all seed stocks or other plant material used. If applicable, state the seed stock centre and catalogue number. If plant specimens were collected from the field, describe the collection location, date and sampling procedures.*

Novel plant genotypes: *Describe the methods by which all novel plant genotypes were produced. This includes those generated by transgenic approaches, gene editing, chemical/radiation-based mutagenesis and hybridization. For transgenic lines, describe the transformation method, the number of independent lines analyzed and the generation upon which experiments were performed. For gene-edited lines, describe the editor used, the endogenous sequence targeted for editing, the targeting guide RNA sequence (if applicable) and how the editor was applied.*

Authentication: *Describe any authentication procedures for each seed stock used or novel genotype generated. Describe any experiments used to assess the effect of a mutation and, where applicable, how potential secondary effects (e.g. second site T-DNA insertions, mosiacism, off-target gene editing) were examined.*
